# Screening for Referral of Serious Pathology by Physical Examination Tests in Patients with Back or Chest Pain: A Systematic Review

**DOI:** 10.3390/ijerph192416418

**Published:** 2022-12-07

**Authors:** Federico Andreoletti, Filippo Maselli, Lorenzo Storari, Andrea Vongher, Monica Erbesato, Marco Testa, Andrea Turolla

**Affiliations:** 1 Departement of Neurosciences, Rehabilitation, Ophthalmology, Genetic and Maternal Infantile Sciences (DINOGMI), University of Genova-Campus of Savona, 17100 Savona, Italy; 2 Department of Human Neurosciences, Sapienza University of Rome, 00185 Rome, Italy; 3 Division of Occupational Medicine, IRCCS Azienda Ospedaliero-Universitaria di Bologna, 40138 Bologna, Italy; 4 Department of Biomedical and Neuromotor Sciences (DIBINEM), Alma Mater Studiorum University of Bologna, 40126 Bologna, Italy

**Keywords:** physical examination, back pain, chest pain, serious pathology, referral

## Abstract

Objective: To investigate the most common physical examination tests (PET) for the screening for referral of patients with back or chest pain caused by serious pathology. Methods: A systematic review was conducted. Searches were performed on seven electronic databases between June 2020 and December 2021. Only studies evaluating patients with back and/or chest pain with clear reporting of PETs and prompt patient referrals were included. Results: 316 full texts were included, and these studies had a total of 474/492 patients affected by a serious disease. Only 26 studies of them described suspicion of serious disease due to at least one positive PET. Cardiac/pulmonary auscultation and heartbeats/blood pressure measurements were the most frequently reported tests. None of the reported studies included physiotherapists and chiropractors who reported the use of various tests, such as: cardiac and pulmonary auscultation, lung percussion, costovertebral angle tenderness, and lymph node palpation, highlighting a lack of attention in measuring vital parameters. On the contrary, doctors and nurses reported the assessment of the range of motion of the thoracolumbar spine and hip less frequently. Conclusions: Appropriate reporting of PETs is sparse, and their utilization is heterogeneous among different healthcare professionals. Further primary studies are needed to describe PETs results in patients suffering from back and/or chest pain.

## 1. Background

In the literature, back pain is an umbrella term used to indicate pain, both chronic or acute, localized in the back within the lumbosacral region (low back pain) and/or in the thoracic spine (upper back pain) [1]. Low back pain is a common condition worldwide as it is experienced by 80% of adults at least once in their lives [2,3]. For upper back pain, the lifetime prevalence ranges from 15.6% to 19.5% in the adult population, and it is an ordinary presentation in primary healthcare clinical practice and the emergency department (ED) [4]. Fortunately, between 85–95% of cases from both conditions are defined as “non-specific” due to uncertain musculoskeletal causes, and they usually have a favourable prognosis that does not require further diagnostic investigations [4,5]. However, low rates of serious diseases mimicking low back pain or upper back pain symptoms are reported, ranging from 1% to 5% depending on the inpatient or outpatient context [6]. These conditions are most often vertebral fractures, spondylodiscitis, cauda equina syndrome, or tumors [7].

Unlike low back pain and upper back pain, chest pain is a term used to describe symptomatology in the thoracic region, both in its anterior and posterior portion, between T1 and T12 on the back and the area of the chest wall from the breastbone to the whole ribs profile on the anterior aspect of the trunk [8,9]. Chest pain represents another reason for access to the ED with a prevalence rate of 25%, and a rough estimate reports that 20–40% of populations complain this symptom at least once during lifetime [10]. About 20% of chest pain events have a non-musculoskeletal cause, which represents a challenge for healthcare professionals; in fact, 50–80% of patients are discharged from EDs without a clear diagnosis [10]. The most frequent pathologies related to primary chest pain are cardiovascular disorders (13.8–16.1%), stable coronary disorders (6.6–11.2%), acute coronary syndrome/myocardial infarctions (1.5–3.6%), lung disorders (10.3–18.2%), chest wall syndrome (24.5–49.8%), and gastrointestinal disorders (5.6–9.7%) [11]. Conversely, musculoskeletal disorders that lead to chest pain symptoms often lack a specific anatomical impairment, and similar to low back pain and upper back pain, chest pain symptoms a commonly diagnosed as non-specific chest pain [12]. 

Collectively, chest pain, upper back pain, and low back pain disorders can be defined as thoracolumbar pain (TLP) disorders [9]. The high incidence within the general population and the high rate of healthcare consultations/ED accesses for TLP raise attention among numerous healthcare professionals involved in the screening for referral of these patients in direct access or primary care settings (i.e., physicians, general practitioners, nurses, physiotherapists, and chiropractors) [2,3,4,10,11,13,14,15,16,17,18]. In the literature, primary care physicians are the professionals who directly evaluate the patients without a referral, and this term commonly labels only general practitioners or medical doctors [14,15,17]. For this reason, in the context of this systematic review, we refer to primary healthcare professionals as all the healthcare professionals listed above who carry out the screening for referral of TLP patients in direct access care settings. This process aims to detect the so-called red flags, namely the signs and symptoms associated with a high risk of serious diseases [3,6,9]. Several published systematic reviews regarding red flags for TLP demonstrated poor accuracy of their utilization if used individually, underpinning the need to combine two or more red flags for a proper referral [6,9,19,20,21,22]. However, most of these reviews largely concern anamnestic evaluation and do not consider physical examination tests (PETs) and their reference values, which have a fundamental role during the common triage process [6,21,23,24]. Our systematic review aims to identify all PETs currently published in the literature for the screening for referral of patients with TLP caused by a severe pathology. Where possible, the positivity of a specific test for a severe pathology is included. The review also aims to report which primary healthcare professional category is accustomed to the use of PETs. This element highlights any gaps in PET utilization by different health professions and considers the need (or a lack of the need) to introduce the most useful PETs in the process of screening for referral of patients with TLP. Finally, our results may help guide the post-graduate education pathways of primary healthcare professionals.

## 2. Methods

This systematic review was conducted according to the guidelines of the PRISMA statement and the Cochrane Collaboration guidelines (Cochrane Handbook 5-1) [25,26]. The protocol was developed using the PRISMA-P framework, and it is registered with the International Prospective Register of Systematic Reviews (PROSPERO), reference: CRD42020192335 [27,28]. The authors of this systematic review have: (a) extensive experience in performing systematic reviews and (b) specific clinical expertise in the screening of patients with TLP. Overall, Cohen’s Kappa (K) was used to quantify the inter-rater agreement between the two authors (F.A., F.M.) for full-text selection, and it was interpreted according to Altman’s definition [29,30].

### 2.1. Eligibility Criteria

#### 2.1.1. Study Designs

Several study designs, including diagnostic accuracy studies, cohort studies, case-control studies, case-series, case-reports, and eventually, randomized controlled trials were considered eligible. Systematic reviews or narrative reviews were excluded. Only studies published in the English, Italian, or Spanish language were included, while no restrictions related to the publication date were applied.

#### 2.1.2. Participants

The studies included must have enrolled patients ≥18 years of age who had been evaluated by a primary healthcare professional for the assessment and/or treatment of TLP (with or without associated symptoms in the upper or lower limbs). Only studies including patients who were assessed using at least one PET and immediately (48 h) or urgent (1 month) sought medical attention or intervention for a suspicion of serious pathology were included, with clear reporting of primary data and subsequent diagnostic confirmation by a gold standard reference test for the specific pathology (e.g., imaging investigations, laboratory tests, surgical procedures, or any other investigation that objectively certifies the presence or the absence of a serious pathology). Furthermore, studies were included only in the case where a serious pathology was confirmed (or not) through diagnostic imaging or any other reference standard procedures. Studies including the primary healthcare professional assessment of patients with a previous diagnosis were excluded unless it was a diagnosis of non-serious pathology. Furthermore, studies were excluded if they evaluated assessments of patients with previous diagnoses of the following diseases and physical health statuses: psychogenic TLP, recent surgery (≤30 days), pregnancy, and tetraparesis (Medical Research Council–MRC = 0/5).

#### 2.1.3. Serious Pathology

In the context of this systematic review, “serious pathology” is an umbrella term that includes malignancy or any other spinal or visceral pathology that is potentially deadly, severely disabling, or needs immediate (48 h) or urgent (30 days) medical attention/intervention. We also included major neuromusculoskeletal pathologies (e.g., spinal and bone fractures, spinal stenosis and radiculopathies requiring surgery within 30 days of assessment, septic spondylitis, etc.) and severe visceral pathologies (e.g., pericarditis, myocardial infarction, abdominal aortic aneurysm, pneumonia, pleuritis, acute diverticulitis, etc.).

#### 2.1.4. Physical Examination Tests (PETs)

In this review, the term “PET” refers to any procedure of observation, palpation, percussion, auscultation, vital signs measurement or any other specific test performed in the outpatient direct access setting for the differential diagnosis. PETs were considered positive in the presence of: (i) clinically significant anatomical impairments (e.g., abdominal mass, Range of Motion (ROM) reduction >25%, or muscle strength reduction ≤3 on MRC scale), and/or (ii) deviation from physiological values (e.g., blood pressure, oxygen saturation, heart rate, breathing rate, body temperature, etc.), and/or (iii) pain or any other significant symptoms or signs in relation to the diagnosed pathology (e.g., painful lymph nodes palpation, palpable pulsatile mass, painful abdominal/chest-wall palpation, positive neurologic or neurodynamic assessment, altered cardiopulmonary auscultation, percussion tests, and altered skin color). Studies, which indicated referrals following a physical examination after one or more treatment sessions (“treat and see” strategy) were also included. In this case, only the tests and values of the last physical examination carried out on the patients were taken into consideration. Studies that reported a differential diagnosis of TLP based only on anamnestic evaluation (with or without the use of questionnaires) or radiographic or laboratory investigations were excluded. For a detailed description of altered reference values for PETs, see Supplementary S1.

### 2.2. Search Methods for Inclusion of Studies—Search Strategy

An electronic search was conducted between April 2020 and December 2021 on the following databases: MEDLINE, EMBASE, CINAHL, The Cochrane Library, PEDro, Web of Science, and Google Scholar. Moreover, Clinical Trials.gov and PROSPERO were searched for ongoing systematic reviews. The search strings were developed according to the PICOTS framework of clinical questions (populations, interventions, comparators, outcomes, timing, and setting) [31]. To make the search strategies sensitive, we did not insert key words for comparisons. The search strategy was developed by a review team using medical subject headings (MeSH) and text words related to “back pain”, “chest pain”, “serious pathology”, “referral”, and “physical examination” and combined with Boolean operators (AND, OR, and NOT). Additionally, we conducted a manual search of all bibliographies of the studies assessed for the subsequent full-text selection. In addition, grey literature was screened (i.e., theses, conference reports, expert opinions, and books) via the Internet. After the MEDLINE strategy was defined, the same strategy was adapted to the syntax and subject headings of the other electronic databases. The full search strategy of each consulted databases, is available in the Supplementary S2.

### 2.3. Studies Selection and Data Extraction

All titles and abstracts were screened independently by two reviewers (F.A. and F.M.). Authors were contacted in order to obtain the full-text paper when necessary. Finally, full texts were independently screened and assessed for eligibility by two of the authors (F.A. and F.M.), while disagreements were solved by a third reviewer (A.T.) who was not involved in the screening and data extraction processes. Two reviewers (F.A. and L.S.) independently collected data from all the eligible papers. Data extraction was organized as follows:

- General information (authors, study design, setting, and number of assessments);
- Participants (age, gender, sample size, diagnostic criteria, comorbidities, pain duration, and structures of recruitment);
- Type of PETs;
- PETs values;
- Primary healthcare professional in charge; and
- Serious pathology diagnosed.

Discrepancies were identified and resolved through discussion with another reviewer (M.E.). Missing data were requested by the authors.

### 2.4. Inter-Rater Agreement

Cohen’s Kappa (K) was used to quantify the inter-rater agreement between the two authors (F.A. and F.M.) for the full-text selection. Cohens’ K was interpreted according to Altman’s definition: 

k < 2 poor, 0.2 < k < 0.4 fair, 0.41 < k < 0.60 moderate, 0.61 < k < 0.80 good, and 0.81 < k < 1.00 excellent.

In this screening phase, agreement between the two authors (F.A. and F.M.) was good (Cohen’s K: 0.76) [29,30].

### 2.5. Risk of Bias

Two reviewers (F.A. and L.S.) independently assessed the quality of the studies using the Joanna Briggs Institute’s critical appraisal tools (https://joannabriggs.org/ebp/critical_appraisal_tools, accessed on 1 February 2022). A specific checklist for each study design (case control studies, cohort studies, diagnostic test accuracy studies, case series, case reports, RCTs, etc.) was used. Discrepancies were resolved by two other reviewers (A.T. and M.T.). Regarding the diagnostic accuracy studies, the QUADAS-2 tool (The University of Bristol, Bristol, UK) [32], was also used in addition to the JBI critical appraisal tool (The University of Adelaide, Adelaide, Australia)

### 2.6. Data Analysis

Qualitative and quantitative analyses were conducted on the data obtained from the studies included in the review. The differences between the studies were summarized in table form. 

Due to the high heterogeneity of the included studies, mainly regarding the study designs, characteristics of patients, and PETs used, no meta-analysis was performed, and qualitative analysis was conducted. The quantitative analysis was conducted using Excel software (Microsoft 2019, version 16.25, Microsoft Corporation, Redmond, WA, USA). Where available, we reported the values and diagnostic accuracy of the involved PETs.

## 3. Results

**Figure 1:**
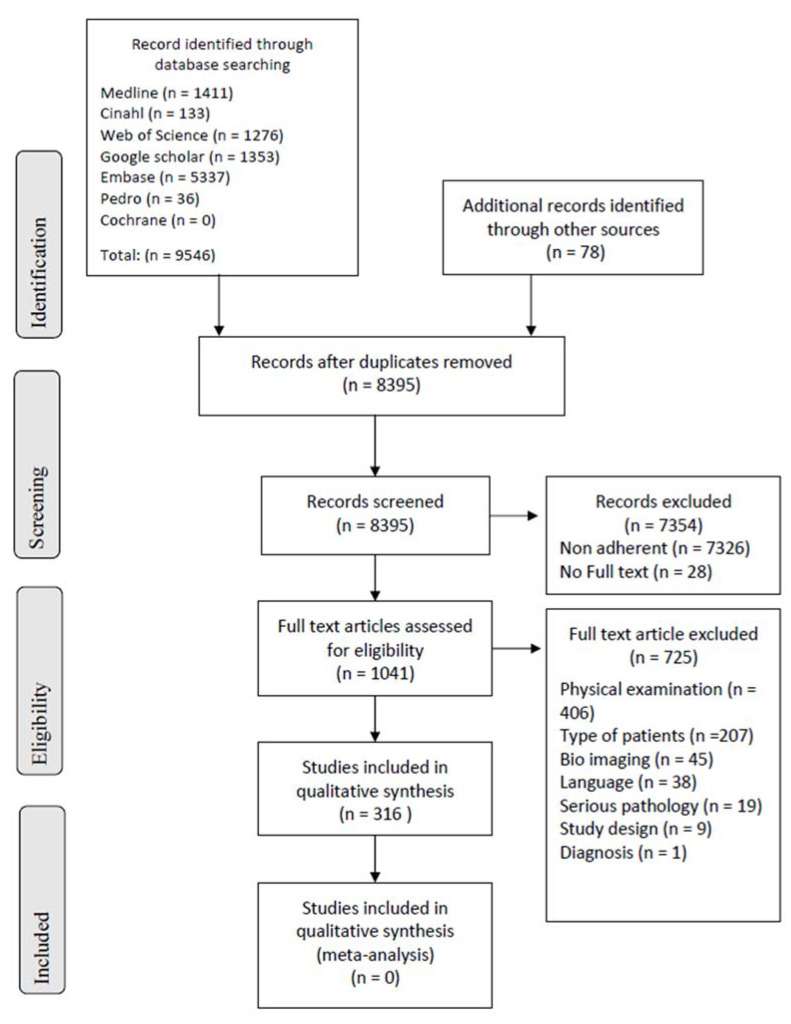
Flow diagram describing the selection process of the articles according to the model indicated by the PRISMA [25].

Electronic search strings identified 9468 records. We also identified a further 78 references through manual searches by cross-reference screening for a total of 9546 eligible records [33]. The removal of 1229 duplicates left 8395 records. Then, we excluded 7329 records reviewing titles and abstracts, and other 25 records were excluded due to the failure of full-text retrieval, thus leaving 1041 studies eligible for full text assessment. Subsequently, 725 full text papers were removed because they did not meet the inclusion criteria. The full search process is reported in Figure 1.

### 3.1. Study Characteristics

#### 3.1.1. Study Designs

In total, 316 articles were included [1a–316a in Supplementary S3]. Of these articles, 298 are case reports, 14 are case series, 1 is a retrospective cohort study without a parallel cohort, and 3 are diagnostic studies. The list of the studies included is reported in Supplementary S3.

#### 3.1.2. Sample

Characteristics of the population included in this review are detailed in Table 1.

A total of 492 patients met the eligibility criteria previously described. It was possible to record the gender (men: 235; women: 169; F/M ratio: 1.39, M: male; F: female) and age (mean age: 48.9 y ± 17.3) of only 404 patients. Of the 492 patients, 243 patients (49.4%) were referred to a primary healthcare professional for low back pain, 117 patients (23.8%) for chest pain, 30 patients (6.1%) for upper back pain, and 102 patients (19.6%) for a combination of two of these symptoms. A final diagnosis of serious pathology was found in 474 patients.

### 3.2. Risk of Bias

**Figure 2:**
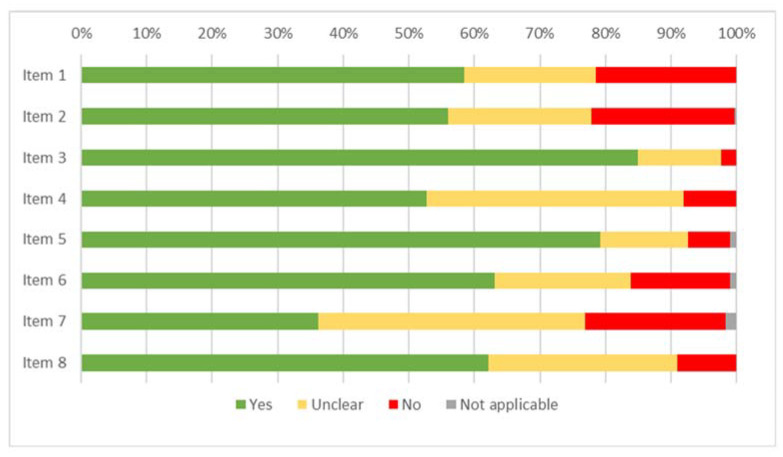
Summary of the critical appraisal process of the case reports according to the JBI tools.

**Figure 3:**
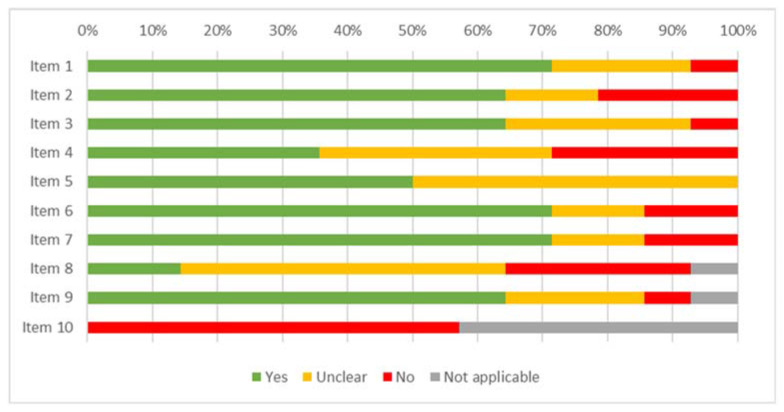
Summary of the critical appraisal process of the case series according to the JBI tools.

**Figure 4:**
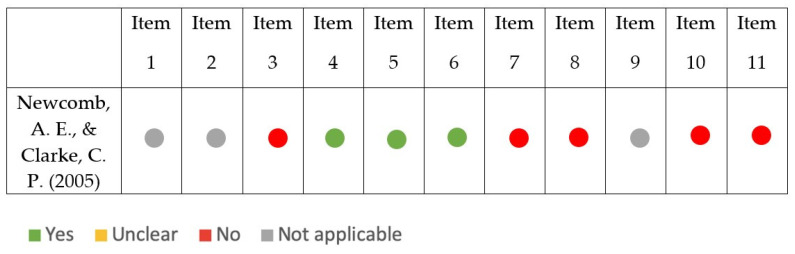
Summary of the critical appraisal process of the cohort studies according to the JBI tools [221a in Supplementary S3].

**Figure 5:**
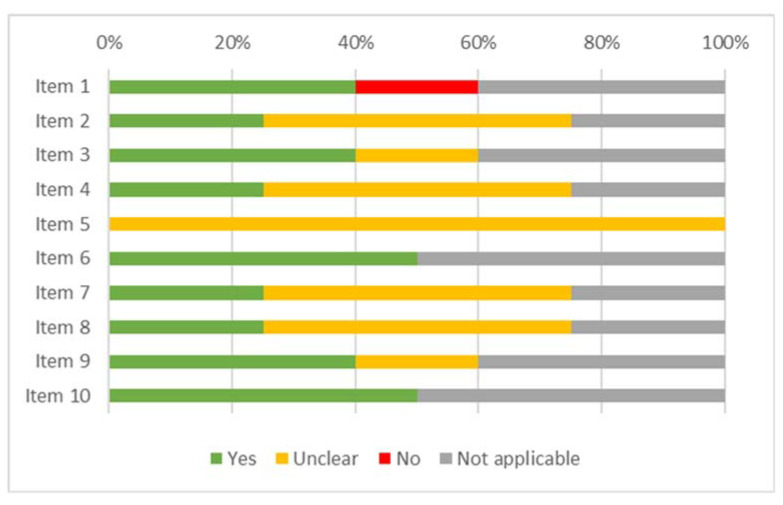
Summary of the critical appraisal process of the diagnostic accuracy of the studies according to the JBI tools.

**Figure 6:**
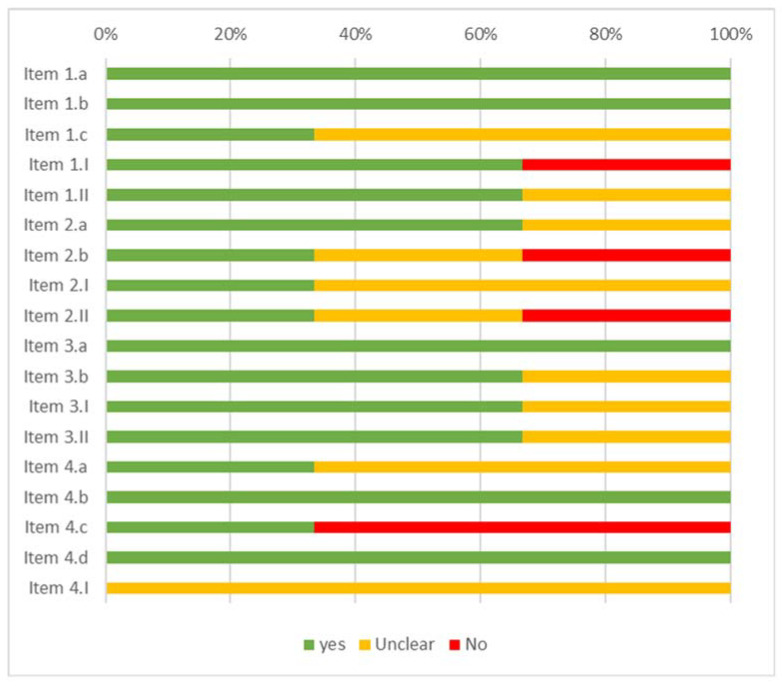
Summary of the critical appraisal process of the cohort studies according to QUADAS 2 [32].

The results of the risk of bias assessment for the studies included in the review are displayed in Figure 2 (case report), Figure 3 (case series), Figure 4 (cohort study), Figure 5 (diagnostic studies by JBI), and Figure 6 (diagnostic studies by QUADAS 2). 47.1% of the case reports showed an inadequate score in “item 4” of the “critical appraisal tools” of the JBI (the one concerning the precision in the description of the diagnostic tests). During the critical appraisal process, the agreement between the two authors was good (Cohen’s K: 0.63) [29].

### 3.3. Synthesis of Results

#### 3.3.1. Serious Pathologies

During the anamnesis, in addition to low back pain, chest pain, or upper back pain, 84 patients also reported symptoms in the upper or lower limbs and/or other symptoms (e.g., dyspnea, cough, nausea, neck pain, headache, shortness of breath, etc.). A diagnosis of serious pathology was confirmed in 96.3% of the patients (474/492), of which 156 (32,9%) were cardiovascular diseases; 33 (7.0%) were pulmonary diseases; 94 (19.8%) were tumors; 96 (20.3%) were fractures; 34 (7.2%) were visceral diseases; 32 (6.8%) were infections; and 29 (6.1%) needed immediate (within 48 h) or urgent (within 30 days) medical intervention for other diseases. Additionally, some patients reported a secondary impairment. A pathological fracture was found in 28 patients (5.9% of the 474 patients diagnosed with serious pathology), while a compression of the spinal cord or cauda equina was found in 34 patients (7.2%) (Table 1).

#### 3.3.2. Physical Examination Test

Cardiac auscultation was the most used PET, which was reported in 149 patients, with positive findings in 118 patients, of which 97.5% had a serious pathology diagnosis (Table 2). Pulmonary auscultation was reported in 114 patients, with altered sounds in 84 cases (95.2% were serious pathologies). Blood pressure measurement was performed on 108 patients (57 positive findings, 94.7% serious pathologies) and heart rate measurement was reported for 101 patients (60 positive cases, 96.7% serious pathologies). PETs, such as cardiac auscultation, pulmonary auscultation, lymph node palpation, pulmonary percussion, costovertebral angle tenderness, body temperature measurement, respiratory rate, and blood oxygen saturation were reported only by physicians or nurses.

#### 3.3.3. Primary Healthcare Professional in Charge

Of the 492 patients included, 197 patients (40.0%) were assessed by a physician, 126 patients (25.6%) were assessed by the ED team, 13 patients (2.7%) were assessed by a physical therapist, and 4 patients (0.8%) were assessed by a chiropractor. For the remaining 152 patients, it was not possible to determine which primary healthcare professional conducted the physical examination (Table 3). For 337 patients (68.5%), the primary healthcare professional in charge was the first professional to assess the TLP signs and symptoms, and in the other cases, it was a second examination.

The blood pressure measurement was evaluated for 13% of the patients by a physician, 50% of the patients by the ED team, 1% of the patients by physiotherapists, 1% of the patients by chiropractors, and 35% of the patients’ blood pressure measurement was evaluated by unspecified professionals. Conversely, the evaluation of the ROM was carried out mostly by physiotherapists and chiropractors both for the spine (reported in 11 patients: 46% physiotherapists; 18% chiropractors; 18% physicians; and 18% unspecified professionals) and the hip (reported in 9 patients: 44% physiotherapists; 11% chiropractors; 33% unspecified professionals; and 11% assessed by the ED team) (Table 3).

A greater heterogeneity was found among professionals who reported the use of abdominal palpation (positive in 18 patients, of which 94.4% were diagnosed with serious pathology), the palpation of the sternum, ribs, vertebral processes, and pelvic girdle (positive in 39 patients, of which 37 had a diagnosis of serious pathology), and the use of neurological examination (osteotendinous reflexes, sensitivity, strength, and straight leg raise tests). However, physicians and ED team professionals did not use the SLUMP test as part of the screening for referral process.

#### 3.3.4. PET Diagnostic Accuracy

The three diagnostic studies included in our systematic review reported the accuracy of four PETs. In particular: Berger et al. (1990) calculated the diagnostic accuracy of cardiac auscultation in patients with myocardial disease (Sensitivity (Sn): 61.6%; Specificity (Sp): 59.0%; Positive Predictive Value: +45.5%; Negative Predictive Value: −73.4%); Grani et al. (2015) described the diagnostic accuracy of palpation of the sterno-costal complex in patients suffering from acute coronary syndrome (Sn: 92.9%; Sp: 48.6%; VPN: 98.1%); and Langdon et al. (2010) reported data of the closed fist percussion test (Sn: 87.5%; Sp: 90%) and of the supine sign (Sn: 81.3%; Sp: 93.3%) and the diagnostic accuracy in patients with vertebral fracture [1a–3a in Supplementary S3].

The positivity of used PETs was clearly reported in only 26 of the 316 included studies. In contrast, in the other included studies, at the end of the clinical evaluation, an instrumental examination was carried out without a clear description of the diagnostic hypotheses which led the primary healthcare professional to further investigate the patients’ symptoms [4a–29a in Supplementary S4]. The bibliographic list of these studies is reported in Supplementary S4.

In these 26 studies, 32 patients (M: 22; F: 10) were evaluated using PETs: 11 by a physiotherapist, 1 by a physician, 8 by an ED team member, 11 by an unspecified healthcare professional, and 1 by a chiropractor.

The most frequently reported PETs were plantar skin reflex (6 patients, 100% final diagnosis of serious pathology); blood pressure measurement (5 patients, 80% was serious pathology); and pulmonary auscultation, heart rate measurement, reflexes evaluation, and sensory assessment (with each test positive for 5 patients, and 100% were serious pathologies as final diagnosis) (Table 4).

## 4. Discussion

To the best of the authors’ knowledge, this is the first systematic review concerning the utilization and diagnostic accuracy of PETs that aimed to identify possible underlying serious pathologies in patients with TLP. 

According to the results from the 316 included studies, we found 492 patients had positive results for at least one PET, of which 96.3% had a final diagnosis of serious disease. These values shed light on the role of PETs in screening for referral and underline their need within a comprehensive diagnostic process.

Among primary healthcare professionals, there is a trend to underestimate the risk of finding a serious pathology during physical examinations of TLP patients because the prevalence of such pathologies is usually low [34]. For this reason, we investigated the reporting of PETs and their diagnostic accuracy in confirmed serious pathology cases. Our systematic review describes how these tests may have an impact on everyday clinical practice. The findings of our research are biased by an overestimation of the prevalence of serious pathologies among TLP patients, mostly due to old and inaccurate data present in the literature [6,35]. To note, recent studies demonstrated that the prevalence of serious diseases changes depending on the type of study designs and the sample of patients included [23]. Thus, there is a need to update the literature references related to the prevalence of serious pathologies in patients complaining of TLP in direct access settings. However, primary healthcare professionals must be careful to correctly interpret possible red flags during patient evaluations since red flags are useful only if they are present in combination and not as a single alarming finding [9,34].

From the analysis of the 316 studies, only 26 clinicians (Supplementary S4) investigated a serious pathology after carrying out PETs. In these cases, the positive results of one or more PETs helped the primary healthcare professional in charge (32.4% physiotherapist, 26.45% primary healthcare professional in the ED team, 5.9% physician, 2.9% chiropractor, and 32.4% unspecified health professional) to deepen the diagnostic investigations. However, even in these 26 studies, we cannot ascertain that the patient referral was exclusively due to the PETs results. In fact, as reported by Ross et al. (2008), the suspicion of serious disease commonly arises from both anamnesis and physical examination results [26a in Supplementary S3]. Indeed, a proper screening for referral includes a combination of anamnestic data and diagnostic tests to increase the diagnostic accuracy of the evaluation [6,21,35,36]. Furthermore, we underline that most studies did not specify which serious pathology was suspected at the end of the assessment. This is in line with the aims of screening for referral, which are related to rule out the suspicion of a serious disease and not to clear a diagnosis [36].

Regarding the other 290 articles, however, it is impossible to determine if the positive results of one or more PETs really influenced the choice of the clinician for the referral. In fact, in these studies, the patient was subjected to further diagnostic investigations, but the diagnostic hypotheses that led the primary healthcare professional to suspect a serious disease were not explicit. An additional consideration from the analysis of the results is that physiotherapists and chiropractors tend to describe the screening for referral process more accurately. Indeed, 70.6% of physiotherapists or chiropractors made the suspicion of serious disease explicit at the end of the physical examination compared to 3.3% of doctors or members of the ED team.

This trend to describe clinical reasoning and consequent diagnostic hypotheses of serious pathology with less precision could be partially explained by the context within which the physical examination takes place. For example, numerous people can access the ED, which led to the elaboration of decision-making protocols based on the severity of the patient’s symptoms [37]. Therefore, the choice to carry out imaging examination to get the diagnosis may be not related to what emerges during the screening for referral process. This fact, together with the rapid accessibility to diagnostic imaging in ED contexts, could explain part of the difference in behavior between the different primary healthcare professionals in charge. 

Our systematic review identifies the PETs currently reported in the literature for the screening for referral of patients with TLP symptoms, thus revealing a difference between the utilization of PET types by each kind of primary healthcare professional. In fact, some PETs are used more frequently by physicians and/or nurses, while others are used mostly by physiotherapists or chiropractors. Interestingly, none of the included studies included physiotherapists or chiropractors who reported the use of cardiac auscultation or pulmonary auscultation in their screening for referral process, which also highlights a lack of attention to measuring vital parameters.

It is possible that, in some cases, unfair reasoning or cognitive mistakes could be developed within the clinical decision-making process, mainly due to a lack of required knowledge or inadequate data collection [38]. Unfortunately, this phenomenon is not frequently reported because of widespread reporting bias within the literature related to the non-publication of inconclusive results. In this regard, the study of Laslett et al. (2000) [19a in Supplementary S3], described how the physical therapist, despite having correctly determined a cardiovascular system involvement, performed only a bicycle test, an evaluation of the lower limbs pulse, and the hip joint range of motion examination; however, he did not report any review-of-systems measurement [39]. The mistake in this example should not be addressed to primary healthcare professionals themselves, but rather to the often concealed issues of outdated professional expertise and a deficient updating of the knowledge needed to properly frame the screening for referral process. The latter, therefore, should be judged as a “system error” in the physical therapists’ clinical practice, and this is a point to improve and empower in the future if the goal is to get a solution to such an issue [38]. Similarly, although with different diagnostic tests, physicians and other primary healthcare professionals in the ED team seem to use spinal or hip joint ROM evaluations less frequently during the screening for referral process. As described by Chen et al. (2002), the ED physician evaluated the lung sounds in a patient that reported low back pain after a fall, but he did not perform an evaluation of the spinal ROM or a spinal springing test [30a in Supplementary S3]. Indeed, tenderness with direct palpation of the spinous process is the most frequent warning sign of patients with spinal infection or vertebral fractures [9,40]. Despite this, Negrini et al. (2001) clearly reported that about 10.4% of general practitioners do not routinely perform spinal palpation during the screening for referral of low back pain patients [41]. Therefore, it seems that each kind of primary healthcare professional tends to use different diagnostic tests related to their own specific fields of knowledge or those in which they are more confident.

It must be highlighted that several papers in the present systematic review were not sufficiently accurate in the description of the PETs used. This reporting bias did not allow us to add them within the data extraction; therefore, our results might be influenced by this factor. In particular, the details of the costovertebral palpation maneuvers were often poorly described, as were vitals evaluations, nervous system examinations (particularly in strength and sensitivity tests), and muscle tone evaluations [31a–41a in Supplementary S3]. 

However, the PETs used to perform physical examinations might be varied not only due to the specific professional skills of each primary healthcare professional in charge, but also due to the population of patients subjected to the screening [23]. All primary healthcare professionals involved in the management of TLP patients should therefore take steps to ensure that their background knowledge is adequate enough to handle all the tests in the present systematic review. Nevertheless, the positive or negative values of the reported PETs are not related to their reliability regarding detecting or discarding the presence of a serious pathology. In fact, in our review, we did not calculate or report the psychometric features of each test. We reported only the diagnostic accuracy of the three cross-sectional studies included that were related to heart sounds auscultation, palpation of the sternal–rib joint complex, and the close fist percussion test and the supine sign [1a–3a in Supplementary S3]. In the literature, the PETs often lack diagnostic accuracy, so their results are not exhaustive enough to rule in or rule out serious pathologies, but these clinical signs may constitute useful information within the screening for referral process if properly matched with anamnestic data [21,35,42]. For these reasons, it would be useful for universities that still do not provide such clinical expertise in their degree programs to fill this gap by reconsidering the contents and the needs of the modern core curriculum for each primary healthcare professional category.

### 4.1. Strengths and Limitations

In this review, we did not define the eligibility criteria to evaluate the quality assessment of the included studies. This strategy led us to evaluate a greater amount of full texts, including case reports, which are frequently excluded in the published systematic reviews related to this topic. Furthermore, we excluded those studies with an unclear description of PETs findings. A limitation is also related to the publication bias within the literature toward our objectives. Indeed, it is likely that only case reports with involvements of serious pathology are published; therefore, our readers should consider this issue. Finally, the sample size and the study design of this study was inadequate for performing a metanalysis and an odd’s ratio calculation related to our findings. For this reason, more cross-sectional studies, prospective studies, and case-control studies of high methodological quality are needed to collect trusted results about the clinical and diagnostic meaning of PETs and to create opportunities to statistically calculate the reliability and diagnostic accuracy of PETs to properly evaluate serious pathologies within the population of patients with TLP symptoms.

### 4.2. Directions for the Future

The continuous aging of the global population in the future may further increase the prevalence of serious pathologies. Therefore, primary healthcare professionals, especially if involved in direct access settings, need to be trained and capable of triaging the typical clinical presentations of such pathologies, which potentially mimic symptoms of most common musculoskeletal disorders [9,42,43,44,45,46]. Certain categories of primary healthcare professionals should further improve their knowledge and clinical reasoning skills to match the necessary knowledge and skills for a proper screening for referral process as currently performed by specific healthcare professionals [42,43,44,45,46]. For these reasons, it would be appropriate for these skills to become a milestone of the core competence of all healthcare professionals, both for bachelor and post-graduate academic paths, and to make these skills mandatory topics in academic programs. Improvements in theoretical and practical knowledge will improve the quality of the pathway of care and daily clinical practice, and specifically, in the case of serious pathologies, it will increase screening for referral skills in order to ensure the most favorable prognosis for patients [9,42,43,44,45,46].

## 5. Conclusions

Our findings show some differences in the assessment of PETs by different primary healthcare professionals.

In their screening for referral, physiotherapists and chiropractors tend not to report the use of various tests, such as cardiac and pulmonary auscultation, lung percussion, costovertebral angle tenderness, and lymph node palpation, indicating a lack of attention toward measuring vital parameters. In contrast, primary healthcare professionals on ED teams, physicians, and nurses tend to report the ROM of the thoracolumbar spine and hip less frequently.

These differences between primary healthcare professionals in terms of using different physical examination tests might be due to various reasons, such as the scopes of practice, their academic backgrounds, and their different skills based on various health systems in various countries around the world.

Our results suggest the need for all primary healthcare professionals of patients presenting with TLP symptoms to acquire the skills necessary for the management of PETs.

## Figures and Tables

**Table 1:** Characteristics of the studies and the sample.

Item	*n*	%	Mean (SD)
Type of study			
Case report	298	94.3	
Case series	14	4.4	
Diagnostic	3	0.9	
Case control	1	0.3	
N patients	492		
Age	429	87.2	48.9 y (±17.3)
Sex			
M	235	54.8	
F	169	39.4	
Pain region			
LBP	243	49.4	
CP	117	23.8	
UBP	30	6.1	
LBP–CP	3	0.6	
CP–UBP	9	1.8	
LBP–UBP	90	18.3	
Other symptoms			
UL	83	16.9	
LL	21	4.3	
Dyspnea	56	11.4	
Cough	39	7.9	
Others	147	29.9	
Serious pathology	474	96.3	
Cardiovascular	156	32.9	
Pulmonary	33	7.0	
Tumor	94	19.8	
Fracture	96	20.3	
Visceral	34	7.2	
Infection	32	6.8	
Other	29	6.1	
Secondary impairment			
Pathological fracture	28	5.9	
Spinal cord compression	34	7.2	

LBP: Low back pain; CP: chest pain; UBP: upper back pain; M: male; F: female; UL: upper limbs; LL: lower limbs; y: years; and d: days.

**Table 2:** Physical examination tests performed by primary healthcare professionals.

Test	*n*	POS	*n* Serious	% Serious	NEG	*n* Serious	% Serious	HPP.									
									%	EE	%	PT	%	CH	%	NS	%
T	73	32	32	100.0	41	40	97.6	3	4.11	32	43.84	0	0.00	0	0.00	38	52.05
Blood pressure	108	57	54	94.7	51	48	94.1	14	12.96	54	50.00	1	0.93	1	0.93	38	35.19
Heartbeat	101	60	58	96.7	41	38	92.7	9	8.91	53	52.48	1	0.99	0	0.00	38	37.62
Respiratory frequency	55	35	34	97.1	20	19	95.0	4	7.27	27	49.09	0	0.00	0	0.00	23	41.82
Saturation	47	24	23	95.8	23	22	95.7	3	6.38	24	51.06	0	0.00	0	0.00	20	42.55
Cardiac auscultation	149	118	115	97.5	31	30	96.8	75	50.34	47	31.54	0	0.00	0	0.00	27	18.12
Pulmonary auscultation	114	84	80	95.2	30	30	100.0	17	14.91	46	40.35	0	0.00	0	0.00	52	45.61
Babinski	26	22	22	100.0	4	4	100.0	4	15.38	6	23.08	1	3.85	0	0.00	15	57.69
Reflex	50	32	31	96.9	18	17	94.4	8	16.00	9	18.00	5	10.00	1	2.00	27	54.00
Sensory	81	49	48	98.0	32	31	96.9	11	13.58	22	27.16	7	8.64	3	3.70	38	46.91
Strength	56	21	20	95.2	35	34	97.1	7	12.50	14	25.00	7	12.50	2	3.57	26	46.43
SLR	35	28	24	85.7	7	7	100.0	6	17.14	7	20.00	8	22.86	2	5.71	13	37.14
SLUMP	3	1	1	100.0	2	2	100.0	0	0.00	0	0.00	2	66.67	1	33.33	0	0.00
Abdominal palpation	55	18	17	94.4	37	37	100.0	10	18.18	19	34.55	4	7.27	2	3.64	20	36.36
Costal/vertebral palpation	46	39	37	94.9	7	7	100.0	14	30.43	9	19.57	4	8.70	1	2.17	18	39.13
Lymph node palpation	14	5	5	100.0	9	9	100.0	2	14.29	2	14.29	0	0.00	0	0.00	10	71.43
Mass	24	24	24	100.0	0	0	0.0	4	16.67	4	16.67	0	0.00	0	0.00	16	66.67
Pulsatile mass	8	8	7	87.5	0	0	0.0	2	25.00	2	25.00	1	12.50	2	25.00	1	12.50
Inspection	52	52	51	98.1	0	0	0.0	2	3.85	29	55.77	0	0.00	1	1.92	20	38.46
Other tests																	
∆ pressure UL	3	3	3	100.0	0	0	0.0	0	0.00	3	100.00	0	0.00	0	0.00	0	0.00
Pulmonary percussion	11	11	10	90.9	0	0	0.0	3	27.27	3	27.27	0	0.00	0	0.00	5	45.45
Anal/rectal examination	11	8	8	100.0	3	3	100.0	0	0.00	4	36.36	1	9.09	0	0.00	6	54.55
Abdominal auscultation	4	4	3	75.0	0	0	0.0	0	0.00	1	25.00	0	0.00	1	25.00	2	50.00
Costovertebral angle	6	6	6	100.0	0	0	0.0	3	50.00	0	0.00	0	0.00	0	0.00	3	50.00
Peripheral pulses	17	7	7	100.0	10	9	90.0	1	5.88	10	58.82	1	5.88	0	0.00	5	29.41
ROM spine	11	4	4	100.0	7	7	100.0	2	18.18	0	0.00	5	45.45	2	18.18	2	18.18
ROM hip	9	6	6	100.0	3	3	100.0	0	0.00	1	11.11	4	44.44	1	11.11	3	33.33
FABER	3	2	2	100.0	1	1	100.0	0	0.00	1	33.33	1	33.33	0	0.00	1	33.33
Bicycle test	2	2	2	100.0	0	0	0.0	0	0.00	0	0.00	2	100.00	0	0.00	0	0.00
Close fist percussion test	45	45	42	93.3	0	0	0.0	42	93.33	0	0.00	0	0.00	0	0.00	3	6.67
Supine sign	41	41	39	95.1	0	0	0.0	30	73.17	0	0.00	0	0.00	0	0.00	0	0.00
Spinal percussion	4	4	4	100.0	0	0	0.0	1	25.00	1	25.00	1	25.00	0	0.00	1	25.00
Psoas test	3	2	2	100.0	1	1	100.0	0	0.00	1	33.33	1	33.33	0	0.00	1	33.33
Subcutaneus emphysema	1	1	1	100.0	0	0	0.0	0	0.00	1	100.00	0	0.00	0	0.00	0	0.00
Pulsus paradoxus	1	1	1	100.0	0	0	0.0	0	0.00	0	0.00	0	0.00	0	0.00	1	100.00
PROM of different joints	1	1	1	100.0	0	0	0.0	0	0.00	0	0.00	0	0.00	0	0.00	1	100.00
Romberg	1	1	1	100.0	0	0	0.0	0	0.00	0	0.00	1	100.00	0	0.00	0	0.00
Tinel’s sign (sciatic nerve)	1	1	1	100.0	0	0	0.0	1	100.00	0	0.00	0	0.00	0	0.00	0	0.00
Mennel’s sign	1	1	1	100.0	0	0	0.0	1	100.00	0	0.00	0	0.00	0	0.00	0	0.00
Sacroiliac compression	1	1	1	100.0	0	0	0.0	0	0.00	0	0.00	0	0.00	1	100.00	0	0.00
Lhermitte’s sign	1	1	1	100.0	0	0	0.0	0	0.00	0	0.00	0	0.00	0	0.00	1	100.00
Tuning fork test	1	1	1	100.0	0	0	0.0	0	0.00	0	0.00	1	100.00	0	0.00	0	0.00
Beevor’s sign	1	1	1	100.0	0	0	0.0	0	0.00	0	0.00	0	0.00	0	0.00	1	100.00
Altered muscle tone	1	1	1	100.0	0	0	0.0	0	0.00	0	0.00	0	0.00	0	0.00	1	100.00
Clonus test	1	0	0	0	1	1	100.0	0	0.00	0	0.00	1	100.00	0	0.00	0	0.00
Breast palpation	1	0	0	0	1	1	100.0	0	0.00	1	100.00	0	0.00	0	0.00	0	0.00

POS: positive; NEG: negative; HPP: healthcare professional physician; EE: emergency equipe; PT: physiotherapist; CH: chiropractor; NS: not specified; T: temperature; SLR: straight leg raise; ∆: difference; UL: upper limb; ROM: range of motion; PROM: passive range of motion; FABER: Flexion Abduction External Rotation.

**Table 3:** Assessment by primary healthcare professionals.

Item	*n*	%
Primary Healthcare Professional		
Physician	197	40.0
Emergency equipped	126	25.6
Physiotherapist	13	2.6
Chiropractor	4	0.8
Not specified	152	30.9
Direct access		
1°	337	68.5
2°	29	5.9
3°	5	1.0
>3°	6	1.2
See and treat	1	0.2
Not specified	114	23.2

The health professionals who assessed the sample of patients included in the review. The number of visits made before the physical examination in which the suspicion of serious pathology emerged.

**Table 4:** Tests that had a positive result in the 26 articles in which a suspicion of serious pathology was specified.

Positive Test	*n*	%
T	4	12.1
Blood pressure	5	15.2
Heartbeat	5	15.2
Respiratory frequency	1	3.0
Saturation	1	3.0
Cardiac auscultation	1	3.0
Pulmonary auscultation	5	15.2
Babinski	6	18.2
Reflex	5	15.2
Sensory	5	15.2
Strength	4	12.1
SLR	3	9.1
Abdominal palpation	3	9.1
Costal/vertebral palpation	2	6.1
Pulsatile mass	1	3.0
Inspection	1	3.0
Other tests		
Pulmonary percussion	1	3.0
Peripheral pulses	4	12.1
ROM spine	2	6.1
ROM hip	3	9.1
Bicycle test	2	6.1
Psoas test	1	3.0
Tuning fork test	1	3.0
Spinal percussion	1	3.0

T: temperature; SLR: straight leg raise; and ROM: range of motion.

## Data Availability

All data are contained within the article or Supplementary Materials.

## Supplementary Materials

The following supporting information can be downloaded at: https://www.mdpi.com/article/10.3390/ijerph192416418/s1; Supplementary S1: Altered reference values for PETs; Supplementary S2: The full search strategy for MEDLINE; Supplementary S3: The list of the all studies included; Supplementary S4: The bibliographic list of 26 studies in which clinicians declared the suspicion of serious disease at the end of the physical examination.
