# Supplementary material for: Screening for Referral of Serious Pathology by Physical Examination Tests in Patients with Back or Chest Pain: A Systematic Review
Source: Int J Environ Res Public Health. 2022 Dec 7;19(24):16418. doi: 10.3390/ijerph192416418 (PMC9779291; doi:10.3390/ijerph192416418)
Supplement: Supplementary file 1 [file ijerph-19-16418-s001.zip › Supplementary S1 for XML.pdf]

Supplementary S1. Altered reference values for PETs

Body Temperature	> 37.5°
Blood Pressure	> 140 mmHG – 90 mmHg or < 90 mmHg -60 mmHg
Heartbeat	>100 bpm or < 50 bpm
Respiratory frequence	>20 respiratory acts or < 12 respiratory acts
Saturation	< 95%
Babinski	Presence of Babinski's sign in the plantar skin reflex
Reflex	DIminished or absent osteotendinous reflexes
Sensory	Markedly altered sensitivity (if the assessor defines the sensitivity deficit as "mild", the test will not be consider positive)
Strength	Muscle strength ≤ 3/5 MRC
SLR test	SLR test causes patient reported symptom
SLUMP test	SLUMP test causes patient reported symptom
Abdominal palpation	Palpation causes pain or detects abnormalities
Costal/Vertebral palpation	Palpation of the sternum, ribs, pelvic girdle, sacrum, or thoracic and/or lumbar vertebrae causes pain
Lymph node palpation	Palpation causes pain or detects abnormalities
Mass	Inspection or palpation detect the presence of an abnormal mass, painful or not
Pulsatile Mass	Inspection or palpation detect the presence of a pulsatile mass, painful or not
Cardiac auscultation	Auscultation detects abnormalities compared to physiological sounds
Pulmunary auscultation	Auscultation detects abnormalities compared to physiological sounds
Inspection	Inspection detects the presence of jaundice, cyanosis, digital clubbing, edema, skin lesions or any alteration that could lead to suspition of a serious pathology
ROM	Presence of patient-referred pain or a deficit > 25% in passive range of motion (PROM) or active range of motion (AROM) of any joint in the body
Pressure difference in UL	Difference > 10 mmHG in measuring the blood pressure of both UL
Pulmonary percussion	Pulmonary percussion produces non-physiological sounds
Anal/Rectal examination	Altereted anal tone
Abdominal auscultation	Auscultation detects abnormalities compared to physiological sounds
Faber test	Faber test causes patient reported symptom
Bicycle test	Bicycle test (or Van Gelderen test) modifies the patient reported symptom or modifies the patient peripheral arterial pulses.
Close fist percussion test	Close fist percussion test causes patient reported symptom
Supine sign	Supine position causes patient reported symptom
Bone percussion	Percussion on a bony proturberance causes patient reported symptom
Tuning fork test	Application of tuning fork on a bony protuberance causes pain
Psoas test	Psoas test causes patient reported symptom
Subcutaneus emphysema	Skin palpation detects a crepitius
Romberg	The patient strongly falters or falls in the first 30 seconds of the test
Tinel's sign	Peripheric nerve palpation causes patient reported symptom
Sacroiliac compression	Sacroiliac joint compression causes patient reported symptom
Altereted muscle tone	Ashworth scale > 1
Mennel's sign	test causes patient reported symptom
Lhermitte's sign	test causes patient reported symptom
Beevor's sign	During the test, only the upper part of the rectus abdominis contracts pulling the navel towards the head.

The values that determined the positivity or negativity of each physical examination test. SLR: Straight Leg Raise; ROM: Range of Motion; PROM: Passive Range of Motion; AROM: Active Range of Motion; MRC: Medical Research council; UL: Upper Limbs; FABER: Flexion Abduction External Rotation.
