# Supplementary material for: Screening for Referral of Serious Pathology by Physical Examination Tests in Patients with Back or Chest Pain: A Systematic Review
Source: Int J Environ Res Public Health. 2022 Dec 7;19(24):16418. doi: 10.3390/ijerph192416418 (PMC9779291; doi:10.3390/ijerph192416418)
Supplement: Supplementary file 1 [file ijerph-19-16418-s001.zip › Supplementary S2 for XML.pdf]

Supplementary S2

The full search strategy for MEDLINE

MEDLINE (Pubmed)

((((((((((("Back Pain"[Mesh]) OR "Low Back Pain"[Mesh]) OR "Chest Pain"[Mesh]) OR "Pelvic Girdle Pain"[Mesh])) OR ((((("Back pain") OR "Low back pain") OR "Chest pain") OR "pelvic girdle pain"))) OR ((((((("vertebral pain") OR "thoracic pain") OR "sacral pain")) OR "backache") OR "sacroiliac pain") OR "pelvic pain"))) OR ((((((("Lumbosacral Region"[Mesh]) OR "Lumbar Vertebrae"[Mesh]) OR "Thoracic Vertebrae"[Mesh]) OR "Spine"[Mesh]) OR "Sacrum"[Mesh])) OR ((((((("lumbosacral region") OR "lumbar vertebrae") OR "thoracic vertebrae") OR "spine") OR "sacrum") OR "lumbar region") OR "thoracic region"))) AND ((((((((((((((((((("serious illness") OR "serious patholog*") OR "serious disease*") OR "red flag*") OR "malignant disease")) OR "malignanc*")) OR "malignant patholog*") OR ("severe patholog*")) OR ("severe disease*")) OR ((((((((("spinal metastasis") OR "vertebral metastasis") OR "spinal cancer") OR "spinal tumor") OR "malignant cord compression") OR "malignant spinal cord compression") OR "spinal cord compression") OR "neoplastic disease") OR "cancer")) OR (((((("Spinal Fractures"[Mesh]) OR "Osteoporotic Fractures"[Mesh]) OR "Fractures, Compression"[Mesh]) OR "Rib Fractures"[Mesh])) OR (((((("spinal fracture") OR "osteoporotic fracture") OR "compression fracture") OR "rib fracture") OR "vertebral fracture") OR "sacral fracture"))) OR (((((("Tuberculosis, Spinal"[Mesh]) OR "Spondylitis"[Mesh])) OR "Discitis"[Mesh])) OR (((((("spinal tuberculosis") OR "spondylitis") OR "discitis") OR "vertebral infection") OR "vertebral osteomyelitis") OR "spinal infection"))) OR ((((((((((("Coronary Artery Disease"[Mesh]) OR "Coronary Disease"[Mesh]) OR "Heart Diseases"[Mesh]) OR "Pulmonary Heart Disease"[Mesh]) OR "Myocardial Ischemia"[Mesh]) OR "Angina Pectoris"[Mesh]) OR "Angina, Unstable"[Mesh]) OR "Angina, Stable"[Mesh]) OR "Myocardial Infarction"[Mesh])) OR (((((((((((((("coronary artery disease") OR "CAD") OR "coronary disease") OR "heart disease") OR "pulmonary heart disease") OR "myocardial ischemia") OR "angina") OR "angina pectoris") OR "stable angina") OR "unstable angina") OR "myocardial infarction")) OR "pericarditis") OR "heart failure"))) OR (((("Lung Diseases"[Mesh] OR "Lung Diseases, Obstructive"[Mesh]) OR "Pneumonia"[Mesh])) OR ((((((((((("lung disease") OR "pneumonia") OR "pulmonary disorders") OR "pulmonary disease") OR "pulmonary infection") OR "lung infection")) OR "bronchopulmonary disease") OR "bronchopulmonary pathology")) OR "pulmonary disorder"))) OR (((((("Aortic Aneurysm, Abdominal"[Mesh]) OR "Aortic Aneurysm, Thoracic"[Mesh]) OR "Aneurysm"[Mesh]) OR "Aortic Rupture"[Mesh])) OR (((("abdominal aortic aneurysm") OR "thoracic aortic aneurysm") OR "aneurysm") OR "aortic rupture"))) OR ((((((("Gastrointestinal Diseases"[Mesh]) OR "Stomach Diseases"[Mesh]) OR "Intestinal Diseases"[Mesh]) OR "Appendicitis"[Mesh]) OR "Peritonitis"[Mesh])) OR (((((("gastrointestinal disease") OR "stomach disease") OR "intestinal disease") OR "gastric disease") OR "appendicitis") OR "peritonitis"))) OR ((((("Kidney Diseases"[Mesh]) OR ("Male Urogenital Diseases"[Mesh] OR "Female Urogenital Diseases"[Mesh])) OR "Kidney Calculi"[Mesh])) OR (((((("kidney disease") OR "urogenital disease") OR "renal disease") OR "kidney stone disease") OR "kidney calculi") OR "renal calculi"))) OR (((((((("Visceral Pain"[Mesh]) OR "Liver Diseases"[Mesh]) OR "Gallbladder Diseases"[Mesh]) OR "Psoas Abscess"[Mesh]) OR "Pancreatic Diseases"[Mesh]) OR "Splenic Diseases"[Mesh])) OR (((((((((("visceral pain") OR "visceral disease") OR "liver disease") OR "hepatic disease") OR "biliary disease") OR "gallbladder disease") OR "psoas abscess") OR "pancreatic disease") OR "splenic disease") OR "obturator abscess")))) AND (((("Referral and Consultation"[Mesh]) OR "Diagnosis, Differential"[Mesh])) OR (((("referral") OR "screening for referral") OR "consultation") OR "differential diagnosis"))) AND (((((((("Physical Examination"[Mesh]) OR ((((((((("clinical examination") OR "physical examination") OR "clinical test") OR "clinical exam") OR "physical test")) OR "physical assessment")) OR "clinical diagnosis")) OR (((((((("Vital Signs"[Mesh]) OR "Blood Pressure Determination"[Mesh]) OR "Heart Rate Determination"[Mesh]) OR "Body Temperature"[Mesh]) OR "Oximetry"[Mesh]) OR "Respiratory Rate"[Mesh])) OR ((((((((((((("vital sign") OR "vital sign assessment") OR "vital sign measurement") OR "blood pressure measurement") OR "blood pressure assessment") OR "pulse rate measurement") OR "pulse rate") OR "body temperature assessment") OR "body temperature measurement") OR "oxygen saturation measurement") OR "oxygen saturation assessment") OR "respiration rate measurement") OR "respiration rate"))) OR (((("Heart Auscultation"[Mesh]) OR "Auscultation"[Mesh])) OR ((("heart auscultation") OR "lung auscultation") OR "pulmonary auscultation"))) OR ((("tuning fork test")

OR "vertebral percussion") OR "patellar pubic percussion test")) OR (((("Neurologic Examination"[Mesh]) OR (("Reflex, Babinski"[Mesh]) OR "Reflex, Abnormal"[Mesh]))) OR ((((((((((((("Neurologic Examination") OR "Neurologic Exam") OR "sensory test") OR "motor function assessment") OR "sensory function assessment") OR "quantitative sensory test") OR "lasegue") OR "slump") OR "slump test") OR "prone knee bend") OR "babinski sign")) OR "reflex assessment"))) OR (((((((((((((("blumberg's sign") OR "rebound tenderness") OR "rebound tenderness test") OR "pinch-an-inch test") OR "pinch-an-inch") OR "rovsing sign") OR "liver palpation") OR "murphy's sign") OR "costovertebral angle tenderness")) OR "heel test") OR "hop test")) OR ((("abdominal aortic aneurysm auscultation") OR "abdominal aortic aneurysm palpation") OR "abdominal auscultation"))) OR ((("lymph node palpation") OR "clinical breast examination") OR "breast palpation"))

Cochrane Library

#1	MeSH descriptor: [Back Pain] explode all trees	4740
#2	MeSH descriptor: [Low Back Pain] explode all trees	3643
#3	MeSH descriptor: [Chest Pain] explode all trees	4888
#4	MeSH descriptor: [Spine] explode all trees	4573
#5	MeSH descriptor: [Lumbosacral Region] explode all trees	441
#6	MeSH descriptor: [Thorax] explode all trees	536
#7	#1 OR #2 OR #3 OR #4 OR #5 OR #6	14302
#8	"serious pathology"	24
#9	"serious illness"	439
#10	"red flag"	58
#11	"malignancy"	6634
#12	#8 OR #9 OR #10 OR #11	7132
#13	MeSH descriptor: [Referral and Consultation] explode all trees	2195
#14	MeSH descriptor: [Diagnosis, Differential] explode all trees	1371
#15	"screening for referral"	5
#16	#13 OR #14 OR #15	3558
#17	MeSH descriptor: [Physical Examination] explode all trees	97374
#18	"clinical examination"	5580
#19	"clinical diagnosis"	5036
#20	"physical assessment"	197
#21	#17 OR #18 OR #19 OR #20	107455
#22	#7 AND #12 AND #16 AND #21	0

Google scholar

"red flag" "physical examination" "referral" "back pain"

CINAHL

("back pain" OR "low back pain" OR "chest pain" OR "pelvic girdle pain" OR "vertebral pain" OR "thoracic pain" OR "sacral pain" OR "backache" OR "sacroiliac pain" OR "pelvic pain" OR "lumbosacral region" OR "lumbar vertebrae" OR "thoracic vertebrae" OR "spine" OR "sacrum") AND ("serious pathology" OR "serious illness" OR "serious disease" OR "red flag" OR "malignant disease" OR "malignancy" OR "malignant pathology" OR "spinal metastasis" OR "vertebral metastasis" OR "spinal cancer" OR "spinal tumor" OR "malignant cord compression" OR "malignant spinal cord compression" OR "spinal cord compression" OR "neoplastic disease" OR "cancer" OR "spinal fracture" OR "osteoporotic fracture" OR "compression fracture" OR "rib fracture" OR "vertebral fracture" OR "sacral fracture" OR "spinal tuberculosis" OR "spondylitis" OR "discitis" OR "vertebral infection" OR "vertebral osteomyelitis" OR "spinal infection" OR "coronary artery disease" OR "coronary disease" OR "heart disease" OR "pulmonary heart disease" OR "myocardial ischemia" OR "angina" OR "angina pectoris" OR "stable angina" OR "unstable angina" OR "myocardial infarction" OR "pericarditis" OR "heart failure" OR "lung disease" OR "pneumonia" OR "pulmonary disorder" OR "pulmonary disease" OR "pulmonary infection" OR "lung infection" OR "bronchopulmonary disease" OR "bronchopulmonary pathology" OR "abdominal aortic aneurysm" OR "thoracic aortic aneurysm" OR

"aneurysm" OR "aortic rupture" OR "gastrointestinal disease" OR "stomach disease" OR "intestinal disease" OR "gastric disease" OR "appendicitis" OR "peritonitis" OR "kidney disease" OR "urogenital disease" OR "renal disease" OR "kidney stone disease" OR "kidney calculi" OR "renal calculi" OR "visceral pain" OR "visceral disease" OR "liver disease" OR "hepatic disease" OR "biliary disease" OR "gallbladder disease" OR "psoas abscess" OR "pancreatic disease" OR "splenic disease" OR "obturator abscess") AND ("referral" OR "differential diagnosis" OR "screening for referral" OR "consultation") AND ("physical examination" OR "clinical examination" OR "clinical test" OR "clinical exam" OR "physical assessment" OR "clinical diagnosis" OR "vital sign" OR "vital sign assessment" OR "vital sign measurement" OR "blood pressure measurement" OR "blood pressure assessment" OR "pulse rate" OR "pulse rate measurement" OR "body temperature assessment" OR "body temperature measurement" OR "oxygen saturation measurement" OR "oxygen saturation assessment" OR "respiration rate measurement" OR "respiration rate" OR "heart auscultation" OR "auscultation" OR "lung auscultation" OR "pulmonary auscultation" OR "tuning fork test" OR "vertebral percussion" OR "patellar pubic percussion test" OR "neurologic examination" OR "neurologic exam" OR "sensory test" OR "motor function assessment" OR "sensory function assessment" OR "quantitative sensory test" OR "lasegue" OR "slump" OR "slump test" OR "prone knee band" OR "babinski sign" OR "reflex assessment" OR "blumberg's sign" OR "rebound tenderness" OR "rebound tenderness test" OR "pinch-an-inch test" OR "pinch-an-inch" OR "rovsing sign" OR "liver palpation" OR "murphy's sign" OR "costovertebral angle tenderness" OR "heel test" OR "hop test" OR "abdominal aortic aneurysm auscultation" OR "abdominal aortic aneurysm palpation" OR "abdominal auscultation" OR "lymph node palpation" OR "clinical breast examination" OR "breast palpation")

Web of Science

TS = (BACK PAIN) OR TS = (LOW BACK PAIN) OR TS = (CHEST PAIN) OR TS = (pelvic girdle pain) OR TS = (LUMBAR REGION) OR TS = (THORACIC REGION) OR TS = (SPINE) AND TS = (SERIOUS PATHOLOGY) OR TS = (SERIOUS ILLNESS) OR TS = (SERIOUS DISEASE) OR TS = (RED FLAG) OR TS = (MALIGNANT DISEASE) OR TS = (MALIGNANCY) OR TS = (MALIGNANT PATHOLOGY) AND TS = (REFERRAL) OR TS = (DIFFERENTIAL DIAGNOSIS) OR TS = (SCREENING FOR REFERRAL) OR TS = (CONSULTATION) AND TS = (PHYSICAL EXAMINATION) OR TS = (CLINICAL EXAMINATION) OR TS = (CLINICAL TEST) OR TS = (CLINICAL EXAM) OR TS = (PHYSICAL ASSESSMENT) OR TS = (CLINICAL DIAGNOSIS)

PEDro

“referral” “back pain”

EMBASE

("back pain" OR "low back pain" OR "chest pain" OR "pelvic girdle pain" OR "vertebral pain" OR "thoracic pain" OR "sacral pain" OR "backache" OR "sacroiliac pain" OR "pelvic pain" OR "lumbosacral region" OR "lumbar vertebrae" OR "thoracic vertebrae" OR "spine" OR "sacrum") AND ("serious patholog*" OR "serious illness" OR "serious disease" OR "red flag" OR "malignant disease" OR "malignanc*" OR "malignant pathology*" OR "spinal metastasis" OR "vertebral metastasis" OR "spinal cancer" OR "spinal tumor" OR "malignant cord compression" OR "malignant spinal cord compression" OR "spinal cord compression" OR "neoplastic disease" OR "cancer" OR "spinal fracture" OR "osteoporotic fracture" OR "compression fracture" OR "rib fracture" OR "vertebral fracture" OR "sacral fracture" OR "spinal tuberculosis" OR "spondylitis" OR "discitis" OR "vertebral infection" OR "vertebral osteomyelitis" OR "spinal infection" OR "coronary artery disease" OR "coronary disease" OR "heart disease" OR "pulmonary heart disease" OR "myocardial ischemia" OR "angina" OR "angina pectoris" OR "stable angina" OR "unstable angina" OR "myocardial infarction" OR "pericarditis" OR "heart failure" OR "lung disease" OR "pneumonia" OR "pulmonary disorder" OR "pulmonary disease" OR "pulmonary infection" OR "lung infection" OR "bronchopulmonary disease" OR "bronchopulmonary pathology" OR "abdominal aortic aneurysm" OR "thoracic aortic aneurysm" OR "aneurysm" OR "aortic rupture" OR "gastrointestinal disease" OR "stomach disease" OR "intestinal disease" OR "gastric disease" OR "appendicitis" OR "peritonitis" OR "kidney disease" OR "urogenital disease" OR "renal disease" OR "kidney stone disease" OR "kidney calculi" OR "renal calculi" OR "visceral pain" OR "visceral disease" OR "liver disease" OR "hepatic disease" OR "biliary disease" OR "gallbladder disease" OR "psoas abscess" OR "pancreatic disease" OR "splenic disease" OR "obturator abscess") AND ("referral" OR

"differential diagnosis" OR "screening for referral" OR "consultation") AND ("physical examination" OR "clinical examination" OR "clinical test" OR "clinical exam" OR "physical assessment" OR "clinical diagnosis" OR "vital sign" OR "vital sign assessment" OR "vital sign measurement" OR "blood pressure measurement" OR "blood pressure assessment" OR "pulse rate" OR "pulse rate measurement" OR "body temperature assessment" OR "body temperature measurement" OR "oxygen saturation measurement" OR "oxygen saturation assessment" OR "respiration rate measurement" OR "respiration rate" OR "heart auscultation" OR "auscultation" OR "lung auscultation" OR "pulmonary auscultation" OR "tuning fork test" OR "vertebral percussion" OR "patellar pubic percussion test" OR "neurologic examination" OR "neurologic exam" OR "sensory test" OR "motor function assessment" OR "sensory function assessment" OR "quantitative sensory test" OR "lasegue" OR "slump" OR "slump test" OR "prone knee band" OR "babinski sign" OR "reflex assessment" OR "blumberg sign" OR "rebound tenderness" OR "rebound tenderness test" OR "pinch-an-inch test" OR "pinch-an-inch" OR "rovsing sign" OR "liver palpation" OR "murphy sign" OR "costovertebral angle tenderness" OR "heel test" OR "hop test" OR "abdominal aortic aneurysm auscultation" OR "abdominal aortic aneurysm palpation" OR "abdominal auscultation" OR "lymph node palpation" OR "clinical breast examination" OR "breast palpation")
