# Supplementary material for: Screening for Referral of Serious Pathology by Physical Examination Tests in Patients with Back or Chest Pain: A Systematic Review
Source: Int J Environ Res Public Health. 2022 Dec 7;19(24):16418. doi: 10.3390/ijerph192416418 (PMC9779291; doi:10.3390/ijerph192416418)
Supplement: Supplementary file 1 [file ijerph-19-16418-s001.zip › Supplementary S3 for XML.pdf]

Supplementary S3

Supplementary S3: list of the included studies

References

1a. Berger, J.P.; Buclin, T.; Haller, E.; VAN Melle, G.; Yersin, B. Right arm involvement and pain extension can help to differentiate coronary diseases from chest pain of other origin: A prospective emergency ward study of 278 consecutive patients admitted for chest pain. *J. Intern. Med.* **1990**, *227*, 165–172. https://doi.org/10.1111/j.1365-2796.1990.tb00138.x.

2a. Gräni, C.; Senn, O.; Bischof, M.; Cippà, P.E.; Hauffe, T.; Zimmerli, L.; Battegay, E.; Franzen, D. Diagnostic performance of reproducible chest wall tenderness to rule out acute coronary syndrome in acute chest pain: A prospective diagnostic study. *BMJ Open* **2015**, *5*, e007442.

3a. Langdon, J.; Way, A.; Heaton, S.; Bernard, J.; Molloy, S. Vertebral compression fractures—New clinical signs to aid diagnosis. *Ann. R. Coll. Surg. Engl.* **2010**, *92*, 163–166. https://doi.org/10.1308/003588410x12518836440162.

4a. Ahmad, I.; Goyal, N.; Bhatt, C.P.; Chufal, K.S. Primary vertebral leiomyosarcoma masquerading as a nerve sheath tumour. *BMJ Case Rep.* **2017**, *2017*, bcr2016217602. https://doi.org/10.1136/bcr-2016-217602.

5a. Ahmed, A.; Garba, I.; Denue, B.A.; Alkali, M.B.; Bakki, B.; Rawizza, H. Catamenial right haemothorax due to endometriosis: Two case reports. *Afr. J. Respir.* **2015**, *10*, 24–26.

6a. Belliveau, D.J.; Weeks, A.C.; McKelvey, R.; Mulvagh, S.L. Cardiogenic Shock in a Healthy Young Woman With Acute Onset Abdominal and Chest Pain. *JACC Case Rep.* **2020**, *2*, 24–27. https://doi.org/10.1016/j.jaccas.2019.11.066.

7a. Boissonnault, W.G.; Thein-Nissenbaum, J.M. Differential Diagnosis of a Sacral Stress Fracture. *J. Orthop. Sports Phys. Ther.* **2002**, *32*, 613–621. https://doi.org/10.2519/jospt.2002.32.12.613.

8a. Cabanelas, N.; Nobre, Â.; Guerra, N.; Gallego, J.; Ferreira, R.; Carvalheiro, C.; Roque, J.; Peres, M.; Siopa, L.; Martins, V.P.; et al. Images in Cardiology after clinical observation—Aortic dissection in Marfan syndrome. *Rev. Port Cardiol.* **2011**, *30*, 735–741.

9a. Campos, I.D.; Salgado, A.; Azevedo, P.; Vieira, C. Dressler's syndrome: Are we underdiagnosing what we think to be rare? *BMJ Case Rep.* **2019**, *12*, e227772.

10a. Christe, G. Hall, T. The screening process of a patient with low back pain and suspected thoracic myelopathy: A case report. *J Man. Manip. Ther.* **2018**, *26*, 11–17.

11a. Crowell, M.S.; Gill, N.W. Medical Screening and Evacuation: Cauda Equina Syndrome in a Combat Zone. *J. Orthop. Sports Phys. Ther.* **2009**, *39*, 541–549. https://doi.org/10.2519/jospt.2009.2999.

12a. Erhard, R.E.; Egloff, B.P. Patient With Metastatic Adenocarcinoma Imitating Lumbar Herniated Nucleus Pulposis. *J. Manip. Physiol. Ther.* **2004**, *27*, 569–573. https://doi.org/10.1016/j.jmpt.2004.10.001.

13a. Gray, J.C. Diagnosis of Intermittent Vascular Claudication in a Patient With a Diagnosis of Sciatica. *Phys. Ther.* **1999**, *79*, 582–590. https://doi.org/10.1093/ptj/79.6.582.

14a. Greene, G. 'Red Flags': Essential factors in recognizing serious spinal pathology. *Man. Ther.* **2001**, *6*, 253–255. https://doi.org/10.1054/math.2001.0423.

15a. Greenwood, M.J.; Erhard, R.E.; Jones, D.L. Differential diagnosis of the hip vs. lumbar spine: Five case reports. *J. Orthop. Sports Phys. Ther.* **1998**, *27*, 308–315.

16a. Harris, K.K.; Delic, J.A.; Nelson, E.O. Epidural and Paraspinal Abscess Presenting as Acute Low Back Pain. *J. Orthop. Sports Phys. Ther.* **2019**, *49*, 482–482. https://doi.org/10.2519/jospt.2019.8456.

17a. Kaufman, R. Early-Onset Multiple Myeloma: An Illustrative Case Report. *J. Manip. Physiol. Ther.* **2007**, *30*, 543–549. https://doi.org/10.1016/j.jmpt.2007.07.002.

18a. Kodaira, M.; Itoh, T.; Koizumi, K.; Numasawa, Y. Giant right coronary artery aneurysm complicated by a fistula to the right atrium, mimicking a ruptured sinus of Valsalva aneurysm. *BMJ Case Rep.* **2018**, *2018*, bcr2018226197. https://doi.org/10.1136/bcr-2018-226197.

19a. Laslett, M. Bilateral buttock pain caused by aortic stenosis: A case report of claudication of the buttock. *Man. Ther.* **2000**, *5*, 227–233. https://doi.org/10.1054/math.2000.0368.

20a. Lyon, M.; Brannam, L.; Ciamillo, L.; Blaivas, M. False positive abdominal aortic aneurysm on bedside emergency ultrasound. *J. Emerg. Med.* **2004**, *26*, 193–196. https://doi.org/10.1016/j.jemermed.2003.08.012.

21a. Martinez, M.; Lee, A.S.; Hellinger, W.C.; Kaplan, J. Vertebral Aspergillus Osteomyelitis and Acute Diskitis in Patients With Chronic Obstructive Pulmonary Disease. *Mayo Clin. Proc.* **1999**, *74*, 579–583. https://doi.org/10.4065/74.6.579.

22a. Maselli, F.; Rossettini, G.; Viceconti, A.; Testa, M. Importance of screening in physical therapy: Vertebral fracture of thoracolumbar junction in a recreational runner. *BMJ Case Rep.* **2019**, *12*, e229987. https://doi.org/10.1136/bcr-2019-229987.

23a. Matsumura, A.; Ayuzawa, S.; Doi, M.; Enomoto, T.; Takeuchi, S.; Yoshii, Y.; Nose, T. Chronic progressive hematomyelia: Case reports and review of the literature. *Surg. Neurol.* **1999**, *51*, 559–563. https://doi.org/10.1016/s0090-3019(97)00442-4.

24a. McAree, B.J.; E. O'Donnell, M.; Boyd, C.; Spence, R.A.; Lee, B.; Soong, C.V. Inferior Vena Cava Thrombosis in Young Adults—A review of two cases. *Ulster Med. J.* **2009**, *78*, 129–133.

25a. Panwar, N.; Purohit, D.K.; Sharma, S.; Chopra, S. Symptomatic Thoracic Intramedullary Arachnoid Cyst: "A Rare Entity" Report of Two Cases with Short Review of Literature. *J. Neurosci. Rural Pract.* **2019**, *10*, 306–311. https://doi.org/10.4103/jnrp.jnrp_246_18.

26a. Ross, M.D.; Elliott, R.L. Thoracic Spine Compression Fracture in a Patient With Back Pain. *J. Orthop. Sports Phys. Ther.* **2008**, *38*, 214–214. https://doi.org/10.2519/jospt.2008.0404.
27a. Roxas, R.; Gallegos, L.; Bailitz, J. Rapid Detection of Aortic Occlusion With Emergency Ultrasonography. *Ann. Emerg. Med.* **2011**, *58*, 21–23. https://doi.org/10.1016/j.annemergmed.2010.09.001.
28a. Stowell, T.; Cioffredi, W.; Greiner, A.; Cleland, J. Abdominal Differential Diagnosis in a Patient Referred to a Physical Therapy Clinic for Low Back Pain. *J. Orthop. Sports Phys. Ther.* **2005**, *35*, 755–764. https://doi.org/10.2519/jospt.2005.35.11.755.
29a. Tzeng, C.-H.; Chen, W.-K.; Lu, H.-C.; Chen, H.-H.; Lee, K.-I.; Wu, Y.-S.; Lee, F.-Y. Challenges in the diagnosis of Boerhaave syndrome. *Medicine* **2020**, *99*, e18765. https://doi.org/10.1097/md.0000000000018765.
30a. Chen, L.-R.; Chen, Z.-X.; Liu, Y.-C.; Peng, L.; Zhang, Y.; Xu, Q.; Lin, Q.; Tao, Y.-M.; Wu, H.; Yin, S.; et al. Pulmonary contusion mimicking COVID-19: A case report. *World J. Clin. Cases* **2020**, *8*, 1554–1560. https://doi.org/10.12998/wjcc.v8.i8.1554.
31a. Aswani, Y.; Anandpara, K.M.; Hira, P. Extension of pancreatic pseudocyst into psoas muscle in a setting of acute pancreatitis. *BMJ Case Rep.* **2015**, *2015*, bcr2014207822. https://doi.org/10.1136/bcr-2014-207822.
32a. Buggage, R.R.; Soudi, N.; Olson, J.L.; Busseniers, A.E. Epithelioid hemangioendothelioma of the lung: Pleural effusion cytology, ultrastructure, and brief literature review. *Diagn. Cytopathol.* **1995**, *13*, 54–60. https://doi.org/10.1002/dc.2840130112.
33a. Kapetanakis, S.; Chourmouzi, D.; Gkasdaris, G.; Katsaridis, V.; Eleftheriadis, E.; Givissis, P. Functional extra-adrenal paraganglioma of the retroperitoneum giving thoracolumbar spine metastases after a five-year disease-free follow-up: A rare malignant condition with challenging management. *Pan Afr. Med. J.* **2017**, *28*, 94. https://doi.org/10.11604/pamj.2017.28.94.13783.
34a. Narváez, J.; Pérez-Vega, C.; Castro-Bohorquez, F.J.; Vilaseca-Momplet, J. Group B streptococcal spondylodiscitis in adults: 2 case reports. *Jt. Bone Spine* **2004**, *71*, 338–343. https://doi.org/10.1016/j.jbspin.2003.05.001.
35a. Abdullah, D.C.; Raghuram, K.; Phillips, C.D.; Jane, J.A.; Miller, B. Thoracic Intradural Extramedullary Capillary Hemangioma. *Am. J. Neuroradiol.* **2004**, *25*, 1294–1296.
36a. Ahmad, M.N.; Uddin, N.; Muzaffar, S.; Adil, S.N.; Tasneem, Z. Extradural extramedullary hematopoiesis: A rare MRI diagnosis with atypical features. *J. Pak. Med Assoc.* **2004**, *54*, 221–223.
37a. Ku, A.; Lachmann, E.; Tunkel, R.; Nagler, W. Neurosarcoidosis of the conus medullaris and cauda equina presenting as paraparesis: Case report and literature review. *Spinal Cord* **1996**, *34*, 116–120. https://doi.org/10.1038/sc.1996.21.
38a. Lang, J.; Chen, L.; Chen, B.; Chen, K.; Liu, A.; Li, J.; Wang, J. Epithelioid angiosarcoma of the spine: A case report of a rare bone tumor. *Oncol. Lett.* **2014**, *7*, 2170–2174. https://doi.org/10.3892/ol.2014.2055.
39a. Tran, L.K.; Gross, L.M.; Hagley, P.; Minkin, R. Pulmonary hypertension in metastatic breast cancer: A case of pulmonary tumour thrombotic microangiopathy. *BMJ Case Rep.* **2019**, *12*, e229715. https://doi.org/10.1136/bcr-2019-229715.
40a. Samdani, A.F.; Garonzik, I.M.; Jallo, G.; Eberhart, C.G.; Zahos, P. Spinal angiolipoma: Case report and review of the literature. *Acta Neurochir.* **2004**, *146*, 299–302. https://doi.org/10.1007/s00701-003-0196-x.
41a. Uehara, M.; Takahashi, J.; Hirabayashi, H.; Kitahara, J.; Kamijyo, T.; Ebara, S.; Kato, H. Hodgkin's disease of the thoracic vertebrae. *Spine J.* **2013**, *13*, e59–e63. https://doi.org/10.1016/j.spinee.2013.03.026.
42a. Abate, E.; Banki, F.; Hagen, J.A.; Klipfel, N. Giant Cell Tumor of the Sternum. *Ann. Thorac. Surg.* **2009**, *88*, 645–647. https://doi.org/10.1016/j.athoracsur.2008.12.059.
43a. Abdallah, A.; Gokcedag, A.; Ofluoglu, A.E.; Emel, E. Non-traumatic myositis ossificans in the lumbar spine. *Am. J. Case Rep.* **2014**, *15*, 421–425.
44a. Abitbol, L.; D'Urzo, A. Fatal aortic dissection in a 37-year-old man: Missed opportunity. *Can. Fam. Physician* **2014**, *60*, 1116–1119.
45a. Abrencillo, R.A.; Khemasuwan, D.; Diaz-Mendoza, J.I. A Young Man with Acute Progressive Respiratory Distress and a Right Inguinal Mass. *Ann. Am. Thorac. Soc.* **2016**, *13*, 970–975. https://doi.org/10.1513/annalsats.201511-763cc.
46a. Acciarri, N.; Paganini, M.; Fonda, C.; Gaist, G.; Padovani, R. Langerhans cell histiocytosis of the spine causing cord compression: Case report. *Neurosurgery* **1992**, *31*, 965–968.
47a. Addesso, L.; Koenig, D.; Huq, N. Lower Extremity Weakness in an Adolescent Female—A Rare Presentation of Ewing Sarcoma. *Ochsner J.* **2018**, *18*, 402–405. https://doi.org/10.31486/toj.18.0039.
48a. Adeniji-Sofoluwe, A.T.; Hafiz, A. Aortic and left pulmonary artery dilatation: An unusual cause of a left hilar mass and lung collapse. *Pan Afr. Med J.* **2014**, *18*, 130. https://doi.org/10.11604/pamj.2014.18.130.2993.
49a. Afonso, C.; Pereira, J.; Gil-Agostinho, A.; Casimiro, C. Spontaneous rupture of an intercostal artery. *BMJ Case Rep.* **2020**, *13*, e233242. https://doi.org/10.1136/bcr-2019-233242.
50a. Ahmad D, Chairman D, Esmadi, M.; Onofrio, J. Endobronchial Tuberculosis in Potter's Rot. *Chest* **2012**, *142*, 244A.
51a. Al Saidi, K.; Malik, S.A.; Albulushi, A.; Moulton, M.; Chatzizisis, Y.S. Left Ventricular Pseudoaneurysm Complicated With Very Late Rupture 5 Years After Myocardial Infarction. *JACC: Case Rep.* **2019**, *1*, 569–572. https://doi.org/10.1016/j.jaccas.2019.08.019.
52a. Al-Sarraf, N.; Doddakula, K.; Moore, D.; McGovern, E. Idiopathic Chronic Constrictive Pericarditis Secondary to Calcified Pericardial Haematoma. *Intern. Med.* **2007**, *46*, 1113–1115. https://doi.org/10.2169/internalmedicine.46.6454.
53a. Alexander J, Meir A, Vrodos N; Yau Y-H. Vertebral Hemangioma. *Spine* **2010**, *35*, E917–-E920.
54a. Ali, A.; Raza, S.H.; Abbas, T.; Ahmad, N., Albahadely, K. Acute myocardial infarction as a first presentation of left atrial myxoma in a young patient. *Eur. Heart J. Cardiovasc.* **2019**, *20*, 296.
55a. Alirhayim, Z.; Qureshi, W.; Shafiq, A.; Hassan, S. Aortic arch variant presenting as an acute ST elevation myocardial infarction. *BMJ Case Rep.* **2013**, *2013*, bcr2012007962. https://doi.org/10.1136/bcr-2012-007962.

56a. Allam, A.; Fergany, A.; Attia, H. Dissection Flap as Bailout Navigator in Complicated Acute Type B Aortic Dissection (Case Presentation). *Eur. J. Vasc. Endovasc. Surg.* **2019**, *58*, e506. https://doi.org/10.1016/j.ejvs.2019.06.1186.
57a. Amiri, L.; Biyyani, R.S.R.S. Hiatal Hernia: An uncommon presentation of chest pain. *Internet J. Internal Med.* **2008**, *8*, 1.
58a. Anderson, R.E.; Wilcox, S.; Miller, E.S. A Rare but Lethal Cause of Chest Pain. *J. Emerg. Med.* **2015**, *49*, 698–702. https://doi.org/10.1016/j.jemermed.2015.06.066.
59a. Tang, W.; Chen, J. Myocardial infarction and 'dye brushes' sign from an atrial myxoma. *Int. J. Card. Imaging* **2020**, *36*, 1–2.
60a. Arul, P.; Varghese, R.G.B.; Ramdas, A. Pleural Tuberculosis Mimicking Inflammatory Pseudotumour. *JCDR* **2013**, *7*, 709.
61a. Aslam, W.; Perez-Guerra, F.; Jebakumar, D.; A. Culver, D.; Ghamande, S. Acute fibrinous organising pneumonia presenting as a cavitary lung lesion and treatment response to azithromycin. *BMJ Case Rep.* **2019**, *12*, e230868. https://doi.org/10.1136/bcr-2019-230868.
62a. Ayan, A.K.; Seven, B.; Orsal, E.; Demirci, E. Evaluation of bone metastasis as the first presentation of hepatocellular carcinoma using 18-fluorodeoxyglucose positron emission tomography-computed tomography. *Indian J. Nucl. Med.* **2013**, *28*, 171–172.
63a. Aydogan, M.; Karatoprak, O.; Mirzanli, C.; Ozturk, C.; Tezer, M.; Hamzaoglu, A. Severe erosion of lumbar vertebral body because of a chronic ruptured abdominal aortic aneurysm. *Spine J.* **2008**, *8*, 394–396. https://doi.org/10.1016/j.spinee.2006.12.001.
64a. Freitas, A.A.; Ferreira, C.; Milner, J.; Ferreira, J.; Marinho, V.; Alves, P.; Jorge, E.; Goncalves, L. P184 Broken heart complications. *Eur. Heart J.* **2020**, *21*, jez319-054.
65a. Bae, S.H.; Hwang, J.H.; Da Nam, B.; Kim, H.J.; Kim, K.-U.; Kim, D.W.; Choi, I.H. Multiple Ewing Sarcoma/Primitive Neuroectodermal Tumors in the Mediastinum. *Medicine* **2016**, *95*, e2725. https://doi.org/10.1097/md.0000000000002725.
66a. Bae, S.Y.; Kim, H.J.; Oh, H.H.; Kwon, M.K.; Lee, J.H.; Park, M.; Sohn, B.S. Multiple Bone Metastases as the First Manifestation of Hepatocellular Carcinoma in Patient with Noncirrhotic Liver. *Case Rep. Oncol. Med.* **2015**, *2015*, 1–5. https://doi.org/10.1155/2015/512849.
67a. Bajaj, S.; Arunavalli, C.; Immamuddin, S. Rare presentation of tuberculosis. *Indian Heart J.* **2018**, *70*, S113. https://doi.org/10.1016/j.ihj.2018.10.340.
68a. Bakan, N.; Camsari, G.; Gur, A.; Ozkan, G.; Bayram, M.; Gorgulu, F.; Urer, N. A 21-Year-Old Male with Productive Cough, Hemoptysis, Chest Pain, and Weight Loss. *Respiration* **2007**, *74*, 706–709. https://doi.org/10.1159/000105539.
69a. Baksi, A.; Gupta, S.; Ray, U.; Ghosh, S. Spontaneous retroperitoneal haemorrhage in a young adult. *BMJ Case Rep.* **2014**, *2014*, bcr-2013. https://doi.org/10.1136/bcr-2013-202361.
70a. Bala, A.; Gnanalingham, S.; Gnanalingham, K. Thrombosis of abdominal aorta precipitating fatal subarachnoid haemorrhage. *BMJ Case Rep.* **2019**, *12*, e227323. https://doi.org/10.1136/bcr-2018-227323.
71a. Balaban B, Taskaynatan, MA, Gencdogan, S.; Ozgul, A.; Tan, A.K.; Kalyon, T.A. Ewing's sarcoma of the pelvic girdle mimicking the unilateral sacroiliitis and lumbar disc syndrome: Case report. *Pain Clin.* **2006**, *18*, 259–262.
72a. Balasubramanian, C.; Rajaraman, G.; Singh, C.; Baliga, D.K. Benign Fibrous Histiocytoma of the Sacrum—Diagnostic Difficulties Facing This Rare Bone Tumor. *Pediatr. Neurosurg.* **2005**, *41*, 253–257. https://doi.org/10.1159/000087484.
73a. Barrett, K.; Miller, M.L.; Wilson, J. Tophaceous Gout of the Spine Mimicking Epidural Infection: Case Report and Review of the Literature. *Neurosurgery* **2001**, *48*, 1170–1173.
74a. Bilen, E.; Bilge, M.; Yasar, A.S.; Kurt, M. A Prominent Pectinate Muscle Mimicking a Pathological Cardiac Mass. *Echocardiography* **2008**, *25*, 662–662. https://doi.org/10.1111/j.1540-8175.2008.00646.x.
75a. Bird-Lake, E. Severe transient left ventricular dysfunction induced by thyrotoxicosis. *Neth. Heart J.* **2011**, *19*, 352–354. https://doi.org/10.1007/s12471-011-0113-5.
76a. Bocchino, M.; Barra, E.; Lassandro, F.; Ranieri, F.; Muto, R.; Rea, G. Primary pleural haemangioendothelioma in an Italian female patient: A case report and review of the literature. *Monaldi Arch. Chest Dis.* **2010**, *73*, 135–139,. https://doi.org/10.4081/monaldi.2010.298.
77a. Boyaci, A.; Boyaci, N.; Tutoglu, A.; Dokumacı, D.S. Spinal epidural abscess in brucellosis. *BMJ Case Rep.* **2013**, *2013*, bcr2013200946. https://doi.org/10.1136/bcr-2013-200946.
78a. Brass, S.D.; Guiot, M.-C.; Albrecht, S.; Glikstein, R.; Mohr, G. Metastatic hemangiopericytoma presenting as an epidural spinal cord lesion. *Can. J. Neurol. Sci. J. Can. Sci. Neurol.* **2004**, *31*, 550–553. https://doi.org/10.1017/s0317167100003796.
79a. Burden, T.; Thompson, C.; Bonanos, E.; Medford, A.R. Lesson of the month 2: Pulmonary embolism in a patient on rivaroxaban and concurrent carbamazepine. *Clin. Med.* **2018**, *18*, 103–105. https://doi.org/10.7861/clinmedicine.18-1-103.
80a. Butticè, S.; Antonino, I.; Giorgio, A.; Valeria, B.; Stefano, P.; Giuseppe, M.; Carlo, M. Xanthogranulomatous Pyelonephritis Can Simulate a Complex Cyst: Case Description and Review of Literature. *Urol. Case Rep.* **2014**, *2*, 113–115. https://doi.org/10.1016/j.eucr.2014.03.007.
81a. Bydon, M.; De la Garza-Ramos, R.; Bettegowda, C.; Suk, I.; Wolinsky, J.P.; Gokaslan, Z.L. En Bloc Resection of a Giant Cell Tumor in the Sacrum via a Posterior-Only Approach Without Nerve Root Sacrifice: Technical Case Report. *Neurosurgery* **2015**, *11* (Suppl. 3), E472–E478.
82a. Byung-June, J.; Seung-Eun, C.; Sang-Ho, L.; Hyeop, J.S.; Suk, P.S. Solitary lumbar osteochondroma causing sciatic pain. *Jt. Bone Spine* **2007**, *74*, 400–401. https://doi.org/10.1016/j.jbspin.2006.04.010.
83a. Cakmak, H.A. Development of chylothorax and chylous ascites in a patient with congestive heart failure. *Turk Kardiyol. Dernegi Arsivi-Archives Turk. Soc. Cardiol.* **2011**, *39*, 495–498. https://doi.org/10.5543/tkda.2011.01447.
84a. Canpolat, U.; Yorgun, H.; Sunman, H.; Evranos, B.; Ateş, A.H.; Karagöz, T.; Aytemir, K.; Oto, A. PP-002 PULMONARY VALVULAR STENOSIS WITH A POSTSTENOTIC DILATATION MIMICKING LEFT HILAR MASS: A CASE REPORT. *Int. J. Cardiol.* **2010**, *140*, S42–42. https://doi.org/10.1016/s0167-5273(10)70147-9.

85a. Cariati, V.P.; Deng, W. Atypical presentation of thoracic spondylodiscitis caused by Streptococcus mitis. *BMJ Case Rep.* **2014**, *2014*, bcr2013200532。 https://doi.org/10.1136/bcr-2013-200532.
86a. Carmody, K.; Asaly, M.; Blackstock, U. Point of Care Echocardiography in an Acute Thoracic Dissection with Tamponade in a Young Man with Chest Pain, Tachycardia, and Fever. *J. Emerg. Med.* **2016**, *51*, e123–e126. https://doi.org/10.1016/j.jemermed.2016.06.046.
87a. Cevik, C.; Nugent, K.; Topkara, V.K.; Otahbachi, M.; Jenkins, L.A. Left circumflex coronary artery to hepatic vein fistula: A case report and brief review of coronary vasculogenesis. *Cardiovasc. Revascularization Med.* **2009**, *10*, 179–182. https://doi.org/10.1016/j.carrev.2008.06.006.
88a. Chan, T.-Y.; Hsieh, C.-C.; Chen, C.-L.; Huang, Y.-Y.; Chuang, C.-C. Case report: Right-sided native endocarditis presenting with multiple abscess formations [care compliant]. *Medicine* **2019**, *98*, e15961. https://doi.org/10.1097/md.0000000000015961.
89a. Chandrashekaran, S.; Karnatovskaia, L.; Festic, E. Recurrent Exudative Pleural Effusion Due to Thoracic Lymphangiomatosis. *Chest* **2013**, *144*, 485A–485A. https://doi.org/10.1378/chest.1702364.
90a. Changstrom, B; Blaker, B; Valle J; Stauffer, B. A prescription for exercise avoidance. *Clin. J. Sport Med.* **2015**, *25*, 36–37.
91a. Chatha HA, Ullah S., Chan F. Thoracic spinal epidural mixed capillary-cavernous haemangioma: Case report and review of literature. *Pak. J. Med. Sci.* **2009**, *25*, 155–158.
92a. Chaudhry, A.A.; Gul, M.; Chaudhry, A.A.; Moore, W. Case 238: Spontaneous Pneumothorax Secondary to Intrapulmonary Necrobiotic Rheumatoid Nodule. *Radiology* **2017**, *282*, 602–608.
93a. Chawla, R.K.; Madan, A.; Madoiya, R.; Chawla, A. Potato swelling of sternum. *Lung India* **2013**, *30*, 219–221,. https://doi.org/10.4103/0970-2113.116234.
94a. Citro R, Panza A, Masiello P; Ravera, A.; Tedesco, L.; Leone, R.; Mirra, M.; Giudice, R.; Iesu, S.; Silvestri, F.; di Benedetto, G.; et al. Intramural aortic hematoma: No flap no warning? *Monaldi Arch. Chest Dis.* **2015**, *76*, 47–49. https://doi.org/10.4081/monaldi.2011.206.
95a. Coman, R.M.; Glover, S.C.; Gjymishka, A. Febrile pleuropericarditis, a potentially life-threatening adverse event of balsalazide—Case report and literature review of the side effects of 5-aminosalicylates. *Expert Rev. Clin. Immunol.* **2014**, *10*, 667–675.
96a. Cui, L.; Suo, Y.; Zhao, Y.; Li, G.; Liu, T. Mid-Ventricular Obstructive Hypertrophic Cardiomyopathy and Apical Aneurysm Mimicking Acute ST-Elevation Myocardial Infarction. *Ann. Noninvasive Electrocardiol.* **2015**, *21*, 98–101. https://doi.org/10.1111/anec.12284.
97a. Cushen, B.; McKeating, A.; Garvey, J.F.; Dodd, J.D.; Mulcahy, H.; Geoghegan, J.; McKone, E.F.; Gallagher, C.G. Pleural effusion arising from a rare pancreatic neoplasm. *Eur. Respir. J.* **2012**, *40*, 1298–1300. https://doi.org/10.1183/09031936.00035312.
98a. Das, A.; Bhattacharya, D.; Datta, S.; Halder, K.C.; Chattopadhyay, S. Primary pulmonary synovial sarcoma: A case report and review of literature. *Int. J. Appl. Basic Med. Res.* **2016**, *6*, 63–65. https://doi.org/10.4103/2229-516x.174019.
99a. Das, D.K.; Choudhury, U. Bilateral secondary Non- Hodgkin's lymphoma of the breasts: A rare case report. *Int. J. Collab. Res. Intern Med. Public Health* **2012**, *4*, 1158–1161.
100a. de Boer, N.J.; Knaap, S.F.; de Zoete, A. Clinical detection of abdominal aortic aneurysm in a 74-year-old man in chiropractic practice. *J. Chiropr. Med.* **2010**, *9*, 38–41.
101a. De Sá Carneiro, A.L.H.; Braulio, R.; Nunes, M.C.P. Echocardiography of a young man with dyspnoea. *Heart* **2015**, *101*, 1317.
102a. Despeyroux-Ewers, M.; Collin, L.; Cognard, C.; Loubes-Lacroix, F.; Manelfe, C. Inflammatory myofibroblastic tumour of the spinal cord: Case report and review of the literature. *Neuroradiology* **2003**, *45*, 812–817. https://doi.org/10.1007/s00234-003-1069-y.
103a. Doctorian, T.; Mosley, W.J.; Do, B. Apical Hypertrophic Cardiomyopathy: Case Report and Literature Review. *Am. J. Case Rep.* **2017**, *18*, 525–528. https://doi.org/10.12659/ajcr.902774.
104a. Dos Anjos, C.S.; Borges, R.; Chaves, A.C.; Fuzita, W.H.; Silva, C.A.M.; de Oliveira, U.G.L. Cystic angiomatosis, pleural effusion and multiple bone lesions mimicking a metastatic malignant neoplasia: A case report. *J. Med. Case Rep.* **2019**, *13*, 265.
105a. Drachman, D.E., Dudzinski, D.M., Moy, M.P.; Fernandez-del Castillo, C.; Chen, J.H. Case 27-2017—A 32-Year-Old Man with Acute Chest Pain. *N. Engl. J. Med.* **2017**, *377*, 874–882.
106a. Dudeney, S.; O'farrell, D.; Bouchier-Hayes, D.; Byrne, J. Extraspinal Causes of Sciatica. *Spine* **1998**, *23*, 494–496. https://doi.org/10.1097/00007632-199802150-00018.
107a. Duman, H.; Bakırcı, E.M.; Karadağ, Z.; Uğurlu, Y.; Bakirci, E.M. Esophageal Rupture Complicated by Acute Pericarditis. *J. Am. Coll. Cardiol.* **2013**, *62*, C120. https://doi.org/10.1016/j.jacc.2013.08.365.
108a. Duman, I.; Aydemir, K.; Tan, A.K.; Dinçer, K.; Kalyon, T.A. An unusual case of osteoid osteoma clinically mimicking sacroiliitis. *Clin. Rheumatol.* **2006**, *26*, 1158–1160. https://doi.org/10.1007/s10067-006-0280-8.
109a. Effoe, V.S.; O'Neal, W.; Santos, R.; Rubinsztain, L.; Zafari, A.M. Pseudo-Wellens syndrome, acute pancreatitis, and an anomalous coronary artery: A case report. *J. Med. Case Rep.* **2019**, *13*, 1–6. https://doi.org/10.1186/s13256-019-2315-1.
110a. Eisenstat, R.S.; Price, D.B.; Rosenthal, A.D.; Schuss, A.L.; Katz, D.S. Thoracic Epidural Castleman's Disease. *Am. J. Roentgenol.* **2002**, *178*, 208–210. https://doi.org/10.2214/ajr.178.1.1780208.
111a. Ekici, M.A.; Özbek, Z.; Kazancı, B.; Guclu, B. Collapsed L4 Vertebral Body Caused by Brucellosis. *J. Korean Neurosurg. Soc.* **2014**, *55*, 48–50. https://doi.org/10.3340/jkns.2014.55.1.48.
112a. El Asri, A.C.; Akhaddar, A.; El Mostarchid, B.; Naama, O.; Sinaa, M.; Oukabli, M.; al Bouzidi, A.; Gazzaz, M.; Boucetta, M. Multiple lytic lesions of the spine: A rare diagnosis of eosinophilic granuloma in an adult: A case report. *Acta Neurochir.* **2010**, *152*, 703–706.

113a. El-Kersh, K.; Gauhar, U.; Saad, M. Atypical presentation of typical carcinoid. *BMJ Case Rep.* **2014**, *2014*, bcr2013202870. https://doi.org/10.1136/bcr-2013-202870.
114a. Er, U.; Yigitkanli, K.; Simsek, S.; Adabag, A.; Bavbek, M. Spinal intradural extramedullary cavernous angioma: Case report and review of the literature. *Spinal Cord* **2006**, *45*, 632–636. https://doi.org/10.1038/sj.sc.3101990.
115a. Erdal, U.; Mehmet, D.; Turkay, K.; A. Mehmet, I.; Ibrahim, N.; Hasan, B. Esophagus perforation and myocardial penetration caused by swallowing of a foreign body leading to a misdiagnosis of acute coronary syndrome: A case report. *J. Med. Case Rep.* **2015**, *9*, 57. https://doi.org/10.1186/s13256-015-0532-9.
116a. Evangelopoulos, M.E.; Koutsis, G.; Andreadou, E.; Potagas, C.; Dimirakopoulos, A.; Sfagos, C. Neuromyelitis Optica Spectrum Disease with Positive Autoimmune Indices: A Case Report and Review of the Literature. *Case Rep. Med.* **2011**, *2011*, 393568. https://doi.org/10.1155/2011/393568.
117a. Faber, M.D.; Narins, R.G.; Stein, J.H. Insidious Renal Failure. *Hosp. Pract.* **1992**, *27*, 159–176.
118a. Fadel R, Ouellette D, Ramo A; Binz, S.; Lanfranco, O.A. Necrotizing Cavitary Lung Mass in Patient with AIDS: A Rare Manifestation of PCP. *Case Rep.* **2019**, *156*, A800
119a. Falavigna, A.; Righesso, O.; Volquind, D.; Salgado, K.B.; Teles, A. Intraosseous sacral paraganglioma with extradural extension: Case report. *Acta Neurochir.* **2009**, *152*, 475–480. https://doi.org/10.1007/s00701-009-0362-x.
120a. Fei, J.; A. Marill, K. ECG phasic voltage changes associated with spontaneous pneumothorax in a patient with vanishing lung syndrome. *BMJ Case Rep.* **2015**, *2015*, bcr2014207498. https://doi.org/10.1136/bcr-2014-207498.
121a. Fernandez L, Yara JD, Arboleda D; Sua, L.F.; Velasquez, M.; Gutierrez, L. A rare case of tuberculosis in ribs and soft tissues of the thorax. *Am. J. Respir. Crit. Care Med.* **2017**, *195*, A6006.
122a. Ferreira, C.; Costa, T.; Marques, A.V. Diffuse alveolar haemorrhage secondary to propylthiouracil-induced vasculitis. *BMJ Case Rep.* **2015**, *2015*, bcr2014208289. https://doi.org/10.1136/bcr-2014-208289.
123a. Fish, D.E.; Middleton, K.; Gluzman, A. Atypical Presentation of Osteomyelitis, Discitis, Epidural, and Iliopsoas Abscess in Diffuse Idiopathic Skeletal Hyperostosis (DISH) Syndrome. *Am. J. Phys. Med. Rehabil.* **2008**, *87*, 960–966. https://doi.org/10.1097/phm.0b013e3181837724.
124a. Fitzpatrick, D.; Longondjo, M. Gastric perforation through a hiatus hernia into the left lung in an 84-year-old woman. *BMJ Case Rep.* **2019**, *12*, e227956. https://doi.org/10.1136/bcr-2018-227956.
125a. Fujimoto, D.; Takami, M.; Kozuki, A.; Shite, J. A case report of unusual clinical features of a spontaneous coronary artery rupture: Pathologic findings in the rupture site. *Eur. Hear. J.—Case Rep.* **2019**, *3*, ytz135. https://doi.org/10.1093/ehjcr/ytz135.
126a. Gaitzsch, J.; Berney, J. Spinal subarachnoid hematoma of spontaneous origin and complicating anticoagulation. Report of four cases and review of the literature. *Surg. Neurol.* **1984**, *21*, 534–538. https://doi.org/10.1016/0090-3019(84)90265-9.
127a. Garcia D.; Young L. Lymphocytic interstitial pneumonia as a manifestation of SLE and secondary Sjogren's syndrome. *BMJ Case Rep.* **2013**, *2013*, bcr2013009598.
128a. Ghazanfari NA, Arif I. Harris D. Triple whammy: A case of type a aortic dissection complicated by coronary artery occlusion and cardiac tamponade. *J. Am. Coll. Cardiol.* **2018**, *71*, A2192.
129a. Gnanalingham K, Bhattacharjee S, O'Neill K. Intraosseous malignant peripheral nerve sheath tumor (MPNST) of the thoracic spine: A rare cause of spinal cord compression. *Spine*. **2004**, *29*, E402–E405.
130a. Gonzva, J.; Viard, F.-V.; Jost, D.; Lefort, H.; Tourtier, J.-P. Transient right bundle branch block in a patient with acute pulmonary embolism. *J. Electrocardiol.* **2016**, *50*, 211–213. https://doi.org/10.1016/j.jelectrocard.2016.10.006.
131a. Gopaul, K.P.; Parry, H.M.; Cullington, D. Twenty-three year-old with pleuritic chest pain. *Heart* **2018**, *105*, 464–469. https://doi.org/10.1136/heartjnl-2017-312718.
132a. Grant, M.; Porter, A.; Stovak, M. Basketball player with dyspnea. *Clin. J. Sport Med.* **2012**, *22*, 198–199.
133a. Grover, H.; Ahluwalia, A.P.; Sethi, S. Imaging Diagnosis of a Rare Presacral Tumour. *Pol. J. Radiol.* **2017**, *82*, 258–262. https://doi.org/10.12659/pjr.900522.
134a. Gül, S.; Kalaycı, M.; Edebali, N.; Yurdakan, G.; Açıkgöz, B.; Acikgoz, B.; Kalayci, M. A multilevel thoracolumbar meningioma in a young woman. *Acta Neurochir.* **2008**, *150*, 843–844. https://doi.org/10.1007/s00701-008-1609-7.
135a. Güzel, E.; Er, U.; Güzel, A.; Toktaş, Z.; Yapıcıer, Ö. Melanotic schwannoma of the L5 root. *Neuroradiol. J.* **2016**, *29*, 219–221. https://doi.org/10.1177/1971400916638359.
136a. Haleem, S.M.; Ahmad, S.; Patel, B. A Rare Cause of Chest Pain. *JACC Case Rep.* **2020**, *2*, 314–315. https://doi.org/10.1016/j.jaccas.2019.09.025.
137a. Hanna, R.R.; Kolanko, N.; Torres, C. Case 234: Neuropathic Spondyloarthropathy. *Radiology* **2016**, *281*, 314–319. https://doi.org/10.1148/radiol.2016132898.
138a. Heckman, A.J.; Mohseni, M.; Villanueva, A.; Cowart, J.B.; Graham, C.G. Concurrent Spontaneous Pneumomediastinum and Pneumorrhachis. *J. Emerg. Med.* **2018**, *54*, e117–e120. https://doi.org/10.1016/j.jemermed.2018.02.036.
139a. Henry, A.P.; Lachmann, E.; Tunkel, R.S.; Nagler, W. Pelvic insufficiency fractures after irradiation: Diagnosis, management, and rehabilitation. *Arch. Phys. Med. Rehabil.* **1996**, *77*, 414–416. https://doi.org/10.1016/s0003-9993(96)90094-5.
140a. Herr, C.H.; Williams, J.C. Supralevator anorectal abscess presenting as acute low back pain and sciatica. *Ann. Emerg. Med.* **1994**, *23*, 132–135. https://doi.org/10.1016/s0196-0644(94)70020-6.
141a. Hilal, T. Diffuse unresolving pulmonary nodules in a patient with a history of cervical carcinoma. *BMJ Case Rep.* **2015**, *2015*, bcr2014208959. https://doi.org/10.1136/bcr-2014-208959.
142a. Hinkamp, C.A.; Dalal, S.N.; Butt, Y.; Chan, A.V.C. Diffuse epithelioid malignant mesothelioma of the pleura presenting as a hydropneumothorax and vertebral body invasion. *BMJ Case Rep.* **2020**, *13*, e231987. https://doi.org/10.1136/bcr-2019-231987.

143a. Hoch, B.; Hermann, G. Migrated herniated disc mimicking a neoplasm. *Skelet. Radiol.* **2010**, *39*, 1245–1249. https://doi.org/10.1007/s00256-010-1004-3.
144a. Hsieh, M.-F.; Wu, I.-W.; Tsai, C.-J.; Huang, S.-S.; Chang, L.-C.; Wu, M.-S. Ureteral Endometriosis with Obstructive Uropathy. *Intern. Med.* **2010**, *49*, 573–576. https://doi.org/10.2169/internalmedicine.49.2797.
145a. Huang, J.; Cohen, S.; Jour, G. Primary small intestine mesenteric low-grade fibromyxoid sarcoma with foci of atypical epithelioid whorls and diffuse DOG1 expression: A case report. *Diagn. Pathol.* **2020**, *15*, 1–7. https://doi.org/10.1186/s13000-019-0905-2.
146a. Hussain, J.; Westerveld, D.; Sattari, M. Cutaneous metastasis: A rare herald of a silent cancer. *BMJ Case Rep.* **2016**, *2016*, bcr2015213734. https://doi.org/10.1136/bcr-2015-213734.
147a. Hussain, R.; Halmai, L.; Subkovas, E. Unexpected sudden death in a young man with myopericarditis--a case report. *J. Ayub Med. Coll. Abbottabad JAMC* **2015**, *27*, 239–240.
148a. Idowu, B.M. Prostate carcinoma presenting with diffuse osteolytic metastases and supraclavicular lymphadenopathy mimicking multiple myeloma. *Clin. Case Rep.* **2017**, *6*, 253–257. https://doi.org/10.1002/ccr3.1336.
149a. Ikeda, S.; Koga, S.; Yamagata, Y.; Koide, Y.; Kawano, H.; Mukae, H.; Mamemura, K. Pulmonary Thromboembolism Caused by Calcification in the Inferior Vena Cava of a Japanese Adult. *Intern. Med.* **2019**, *58*, 1907–1912. https://doi.org/10.2169/internalmedicine.2259-18.
150a. Ilgi, M.; Kutsal, C.; Albayrak, A.T.; Kirecci, S.L.; Temel, U. A rare malformation of urinary system: Right ectopic thoracic kidney. **2017**, *9*, 299–300. https://doi.org/10.4103/ua.ua_169_16.
151a. Jacinto, N.; Madaleno, C.; Rocha, P.; Santos, J.M. Microscopic polyangiitis as an early manifestation of pancreatic cancer. *Eur. J. Intern. Med.* **2013**, *24*, e154. https://doi.org/10.1016/j.ejim.2013.08.399.
152a. Jayanna, M.B.; Schima, S.; Aboeta, A. Left Unilateral Pulmonary Edema Complicating Acute Myocardial Infarction: A Rare Presentation. *Chest* **2012**, *142*, 105A. https://doi.org/10.1378/chest.1389479.
153a. Jia, L.; Ji, H.; Ren, S.; Li, L.; Li, G.; Zhang, X.; Ding, X. Giant arteriovenous malformation in the conus medullaris mimicking a tumor. *Neurol. India* **2016**, *64*, 823–825,. https://doi.org/10.4103/0028-3886.185406.
154a. Joshi, T.N. Candida albicans spondylodiscitis in an immunocompetent patient. *J. Neurosci. Rural Pract.* **2012**, *3*, 221–222. https://doi.org/10.4103/0976-3147.98261.
155a. Kahanov, L.; Daly, T. Bilateral Pulmonary Emboli in a Collegiate Gymnast: A Case Report. *J. Athl. Train.* **2009**, *44*, 666–671. https://doi.org/10.4085/1062-6050-44.6.666.
156a. Kalluri, S; Crossan, J; Iqbal, Q; Sangwan, Y. A rare case of bronchiectasis. *Am. J. Respir. Crit. Care Med.* **2019**, *199*, A6453.
157a. Kalra, A.; Fabius, D.; Gajera, M.; Palaniswamy, C. Triad of Interstitial Pneumonia, Eosinophilia, and Eosinophilic Pleural Effusion: A Rare Paraneoplastic Manifestation of Lung Adenocarcinoma. *Chest* **2010**, *138*, 101A–101A. https://doi.org/10.1378/chest.10168.
158a. Kampolis, C.F.; Damaskos, C.; Loukeri, A.A.; Garmpis, N., Garmpi, A.; Tomos, I.; Spartalis, E.; Dimitroulis, D.; Patsouras, A.; Douskou, M., et al. Primary Hemangiopericytoma Arising in Extralobar Pulmonary Sequestration: A Coincidence or Two Rare Disorders? *In Vivo* **2019**, *33*, 2141–2145.
159a. Kapoor, V.; Theruvil, B.; Foria, V.; Sampson, M.; Harley, J.M. Vertebral Collapse in a 71-Year-Old Woman. *Clin. Orthop. Relat. Res.* **2004**, *423*, 291–298. https://doi.org/10.1097/01.blo.0000128972.35014.ea.
160a. Karakaş, M.; Buyukkaya, E.; Akcay, A.; Sen, N.; Bilen, P.; Gungor, M.; Kurt, M. PP-337 Multiple Thromboemboli In An Old Woman With Acute Myocardial Infarction. *Int. J. Cardiol.* **2012**, *155*, S210–S211. https://doi.org/10.1016/s0167-5273(12)70510-7.
161a. Karataş, M.; Başaran, C.; Ozgül, E.; Tarhan, C.; Ağildere, A.M. Postpartum sacral stress fracture: An unusual case of low-back and buttock pain. *Am. J. Phys. Med. Rehabil.* **2008**, *87*, 418–422.
162a. Katalinic, D.; Anić, B.; Štern-Padovan, R.; Mayer, M.; Sentic, M.; Cikes, N.; Žarković, K.; Dotlic, S.; Plestina, S. Low back pain as the presenting sign in a patient with primary extradural melanoma of the thoracic spine—A metastatic disease 17 Years after complete surgical resection. *World J. Surg. Oncol.* **2011**, *9*, 150. https://doi.org/10.1186/1477-7819-9-150.
163a. Katz, H.; Rose, C.; Rivera, N.T.; Bray, N. HIV-associated primary effusion lymphoma presenting as a paracardial mass. *BMJ Case Rep.* **2015**, *2015*, bcr2014208718. https://doi.org/10.1136/bcr-2014-208718.
164a. Kaur, R.A. Co-morbidity of low back pain and abdominal aortic aneurysm: A case report. *Clin. Chiropr.* **2004**, *7*, 67–72. https://doi.org/10.1016/j.clch.2004.02.005.
165a. Kazama, I.; Nakajima, T. Apparent ST elevation in right bundle branch block pseudo-mimicking myocardial infarction. *SAGE Open Med. Case Rep.* **2019**, *7*, 2050313X19827748. https://doi.org/10.1177/2050313x19827748.
166a. Kemp, G.L. Value of treadmill stress testing in variant angina pectoris. *Am. J. Cardiol.* **1972**, *30*, 781–783. https://doi.org/10.1016/0002-9149(72)90155-5.
167a. Khan, F.; Srirangan, S.; El-Miedany, Y.; Madaan, S. Widespread lytic lesions-A metastatic or vasculitic process? *Int. J. Surg. Case Rep.* **2016**, *18*, 18–20.
168a. Khan, F.Y.; El-Hiday, A.H.; A. Kamel, H. Typhoid osteomyelitis of the lumbar spine. *Hong Kong Med. J.* **2006**, *12*, 391–393.
169a. Kirschner, A.N.; Kuhlmann, E.; Kuzniar, T.J. Eosinophilic Pleural Effusion Complicating Allergic Bronchopulmonary Aspergillosis. *Respiration* **2011**, *82*, 478–481. https://doi.org/10.1159/000323617.
170a. Kobayashi, H.; Hara, J.; Kitahama, Y.; Hanakita, J. Osteoma Arising From the Lumbar Articular Process-Case Report-. *Neurol. Medico-Chirurgica* **2006**, *46*, 508–511. https://doi.org/10.2176/nmc.46.508.
171a. Kobayashi, Y.; Kobayashi, Y.; Hirohata, A. Left main coronary thrombotic occlusion due to secondary polycythemia in a normal sinus of valsalva. *J. Invasive Cardiol.* **2013**, *25*, 421–422.

172a. Köksal, D.; Bayiz, H.; Gülgösteren, M.; Başay, N.; Mutluay, N.; Boyaci, E.; Berktaş, B.; Çakir, E.; Berkoğlu, M. Recurrence of thymoma after 11 years presenting as diffuse pleural thickening. *Tuberk Toraks* **2012**, *60*, 62–65. https://doi.org/10.5578/tt.1993.
173a. Kooblall, M. An unusual foreign body. *Arch. Bronconeumol.* **2016**, *52*, 116–117.
174a. Kornmann, V.; Van Rijn, P.; Mulder, D.; Reijnders, K. Delayed diagnosis of angiosarcoma of the spleen: Clinically presenting as recurrent haemoperitoneum following embolisation. *BMJ Case Rep.* **2015**, *2015*, bcr2014208956. https://doi.org/10.1136/bcr-2014-208956.
175a. Kozij, N.K.; Thenganatt, J. Connect the dots: A rare case of spontaneous haemopneumothorax. *Thorax* **2013**, *69*, 298. https://doi.org/10.1136/thoraxjnl-2013-204397.
176a. Ksienski, D.; Malhotra, S.; Walker, B.; Levy, R.D. Chest Pain and Cough in a 33-Year-Old Postpartum Woman. *Chest* **2007**, *132*, 1389–1392. https://doi.org/10.1378/chest.07-0480.
177a. Kucharczyk-Foltyn, A.; Sniezek-Maciejewska, M.; Tomala, I.; Dymek, M.; Sadowski, J.; Małecka, B.; Lelakowski, J.; Janion, M. Submassive pulmonary embolism as a mask of acute coronary syndrome. *Cardiol. J.* **2007**, *14*, 402–406.
178a. Kul, S. A hidden cause of hemoptysis: Coronary artery-to-pulmonary parenchyma fistula. *Turk Kardiyol. Dernegi Arsivi-Archives Turk. Soc. Cardiol.* **2011**, *39*, 686–689. https://doi.org/10.5543/tkda.2011.01693.
179a. Kulcu, D.G.; Naderi, S. Differential diagnosis of intraspinal and extraspinal non-discogenic sciatica. *J. Clin. Neurosci.* **2008**, *15*, 1246–1252. https://doi.org/10.1016/j.jocn.2008.01.017.
180a. Kumar, A.; Paswan, S.S.; Prashad, R.; Kumari, R.; Kumar, B. Intrapericardial gossypiboma: Rare cause of intrathoracic mass. *Int. J. Surg. Case Rep.* **2018**, *47*, 75–79. https://doi.org/10.1016/j.ijscr.2018.04.024.
181a. Kwatra, K.; Paul, P.M.; Calton, N.; George, S. Mullerian choristoma in a case of spinal dysraphism. *Indian J. Pathol. Microbiol.* **2015**, *58*, 229. https://doi.org/10.4103/0377-4929.155326.
182a. Kwon, O.K.; Lee, M.G.; Kim, H.S.; Park, M.S.; Kwak, K.M.; Park, S.Y. Invasive Pulmonary Aspergillosis after Influenza A Infection in an Immunocompetent Patient. *Tuberc. Respir. Dis.* **2013**, *75*, 260–263. https://doi.org/10.4046/trd.2013.75.6.260.
183a. Lange, R.A.; Hillis, L.D. Acute Pericarditis. *NEJM* **2004**, *351*, 2195–2202.
184a. Lee, C.K.; Baliski, C.; Zetler, P.; Lee, C.H. Retroperitoneal extralobar pulmonary sequestration mimicking a pheochromocytoma. *Histopathology* **2008**, *52*, 525–527. https://doi.org/10.1111/j.1365-2559.2008.02944.x.
185a. Lee, D.H.; Choi, Y.H. Spontaneous intramedullary hematoma initially mimicking myocardial infarction. *Am. J. Emerg. Med.* **2014**, *32*, 1294.e3–1294.e4. https://doi.org/10.1016/j.ajem.2014.03.002.
186a. Lee, S.; Lee, J.; Hatic, H.; Garimella, P. Proof Is in the Yolk a Unique Case of Anterior Mediastinal Wideningfrom Yolk Sac Tumor. *Am. J. Respir. Crit. Care Med.* **2019**, *199*, A6951**.** https://doi.org/10.1164/ajrccm-conference.2019.199.1_meetingabstracts.a6951.
187a. Li, P.; James, S.; Evans, N.; Davies, A.; Herron, B.; Sumathi, V. Paraganglioma of the cauda equina with subarachnoid haemorrhage. *Clin. Radiol.* **2007**, *62*, 277–280. https://doi.org/10.1016/j.crad.2006.10.004.
188a. Li, Y.; Zhang, H.-Z.; She, L.; Wang, X.-D.; Dong, L.; Xu, E.; Wang, X.-D. Primary extramedullary spinal melanoma mimicking spinal meningioma: A case report and literature review. *Oncol. Lett.* **2014**, *8*, 339–344. https://doi.org/10.3892/ol.2014.2099.
189a. Li, Z.; Deng, Y.; Li, Z.; Wang, T.; Gao, J.; Zhou, W.; Li, Y.; Wang, Y. Primary epidural hemangiopericytoma of the thoracic spine: Case report and literature review. *J. Clin. Neurosci.* **2019**, *60*, 142–147.
190a. Lim, L.; Lee, C.; Abisheganaden, J. Spontaneous massive haemothorax secondary to a bleeding intra-thoracic neural tumour-a case report. *Am. J. Respir. Crit. Care Med.* **2015**, *191*, A3601.
191a. Lima, J.S.; Sandler, B.; McWilliams, E. Chest pain, dyspnoea and elevated D-dimer in a recent air traveller. *BMJ Case Rep.* **2011**, *2011*, bcr0420114104. https://doi.org/10.1136/bcr.04.2011.4104.
192a. Littrell, L.A.; Inwards, C.Y.; Rose, P.S.; Wenger, D.E. Chondrosarcoma arising within synovial chondromatosis of the lumbar spine. *Skelet. Radiol.* **2019**, *48*, 1443–1449. https://doi.org/10.1007/s00256-019-03170-8.
193a. Liu, B.; Qu, L.; Zhu, J.; Yang, Z.; Yan, S. Histoplasmosis mimicking metastatic spinal tumour. *J. Int. Med. Res.* **2017**, *45*, 1440–1446. https://doi.org/10.1177/0300060517708530.
194a. Liu, J.K.; Kan, P.; Schmidt, M.H. Diffuse large B-cell lymphoma presenting as a sacral tumor. *Neurosurg. Focus* **2003**, *15*, 1–5. https://doi.org/10.3171/foc.2003.15.2.10.
195a. Liu, J.X.; Shiau, M.C.; Nonaka, D. An 80-Year-Old Man With Shortness of Breath and Large Right-Sided Pleural Effusion. *Chest* **2010**, *138*, 1247–1252. https://doi.org/10.1378/chest.09-2439.
196a. Lu, Y.-P.; Yao, M.; Zhou, X.-Y.; Huang, B.; Qi, W.-B.; Chen, Z.-H.; Xu, L.-S. False esophageal hiatus hernia caused by a foreign body: A fatal event. *World J. Gastroenterol.* **2014**, *20*, 14510–14514. https://doi.org/10.3748/wjg.v20.i39.14510.
197a. Maamar, M.; El Quessar, A.; El Fatemi, N.; El Hassani, M.R.; Chakir, N.; Jiddane, M. Intramedullary tuberculoma: A case report. *Radiography* **2007**, *13*, 251–254. https://doi.org/10.1016/j.radi.2006.04.006.
198a. Madan, K.; Mittal, S.; Jain, A.; Arava, S.; Hadda, V.; Mohan, A.; Guleria, R. A 26-year-old man with dyspnea and chest pain. *Lung India* **2017**, *34*, 562–566. https://doi.org/10.4103/lungindia.lungindia_111_17.
199a. Mainali, N.R.; Aryal, M.R.; Pradhan, R.; Hope, E.J. An unusual cause of recurrent chest pain in an adult woman: A case of recurrent stress-induced (Takotsubo) cardiomyopathy. *BMJ Case Rep.* **2013**, *2013*, bcr2013009546. https://doi.org/10.1136/bcr-2013-009546.
200a. Majumder, E.; Ayanian, S. Paraneoplastic Cushing's Syndrome in a hypotensive, hypokalemic patient. *Hosp. Med.* **2018**, April *697*, 8-11, .
201a. Malik, S.A.; Malik, S.; Dowsley, T.F.; Singh, B. Left Ventricular Thrombus as a Complication of Clozapine-Induced Cardiomyopathy: A Case Report and Brief Literature Review. *Case Rep Cardiol.* **2015**, 1–5.

202a. Mallick, I.H.; Syed, S.A.; Kar, A.K. Liver rupture following delivery: HELLP needed. *Emerg. Med. J.* **2007**, *24*, 372. https://doi.org/10.1136/emj.2006.044735.
203a. Marinella, M.A. Electrocardiographic manifestations and differential diagnosis of acute pericarditis. *Am. Fam. Physician* **1998**, *57*, 699.
204a. Mawer, D.; Parry, D.; Logan, J.; Rajgopal, A. Anaerococcus lactolyticus causing a mycotic aortic aneurysm: Category: Clinical lesson. *J. Infect.* **2011**, *63*, e18.
205a. Mayfield, J.J.; Goldschlager, N. Woman With Sharp Chest Discomfort. *Ann. Emerg. Med.* **2019**, *74*, 777–781. https://doi.org/10.1016/j.annemergmed.2019.04.017.
206a. McKaigney, C. Hepatic Abscess: Case Report and Review. *WestJEM 21.2 March Issue* **2013**, *14*, 154–157. https://doi.org/10.5811/westjem.2012.10.13268.
207a. Mechelli, F.; Preboski, Z.; Boissonnault, W. Differential Diagnosis of a Patient Referred to Physical Therapy With Low Back Pain: Abdominal Aortic Aneurysm. *J. Orthop. Sports Phys. Ther.* **2008**, *38*, 551–557. https://doi.org/10.2519/jospt.2008.2719.
208a. Melson, E.; Amir, S.; Shepherd, L.; Kauser, S.; Freestone, B.; Kempegowda, P. Myocardial Infarction with non-obstructed coronaries—Atypical presentation of pheochromocytoma. *Endocrinol. Diabetes Metab. Case Rep.* **2019**, *2019*, EDM190089. https://doi.org/10.1530/edm-19-0089.
209a. Mewara, A.; Sharma, K.; Dhillon, M.; Sharma, M. Molecular diagnosis of psoas abscess secondary to Pott's spine. *Clin. Microbiol. Newsl.* **2013**, *35*, 92–93. https://doi.org/10.1016/j.clinmicnews.2013.05.002.
210a. Minoğlu, M.; Akkol, İ.; Özdemir, N.; Yıldırım, L. Co-existence of L5-S1 disc herniation and conus medullaris ependymoma. *Int. J. Surg. Case Rep.* **2014**, *5*, 998–1000.
211a. Mohammed, S.; Kollu, V.S. Rare case of *Propionibacterium acnes*-related splenic abscess. *BMJ Case Rep.* **2018**, *2018*, bcr2018225858. https://doi.org/10.1136/bcr-2018-225858.
212a. Mohanan, S.; Sajeev, C.; Muneer, K.; Rajesh, G.; Krishnan, M.; Pillai, V. An unusual cause of acute cor pulmonale--significance of the 'continuous diaphragm sign'. *Indian Heart J.* **2014**, *66*, 543–545,. https://doi.org/10.1016/j.ihj.2014.08.005.
213a. Morales, J.; Giraldo, M. Case report: Apical variant hypertrophic cardiomyopathy simulating an acute inferior myocardial infarction. *J. Electrocardiol.* **2018**, *52*, 35–37. https://doi.org/10.1016/j.jelectrocard.2018.11.003.
214a. Mukherjee, S.; Akmal, S.; Thakur, B.; Prasad, V.; Yeh, J. Mid-Thoracic Epidural Cavernous Hemangioma Presenting as Subacute Paraparesis. *Neurosurg. Q.* **2014**, *24*, 67–69. https://doi.org/10.1097/wnq.0b013e31828cbf23.
215a. Mukherjee, S.; Kalla, S.; Kumar, A. Post-partum aortic dissection. *J. Obstet. Gynaecol.* **2005**, *25*, 388–390. https://doi.org/10.1080/01443610500135255.
216a. Joshi, J.M.; Munot, M.N.; Utpat, K.V.; Desai, U.D. Malignant mesothelioma—Report of two cases with different presentations. *Indian J. Occup. Environ. Med.* **2019**, *23*, 93–96. https://doi.org/10.4103/ijoem.IJOEM_237_18.
217a. Nakazawa, Y.; Morita, N.; Chikazawa, I.; Miyazawa, K. Ureterosciatic hernia treated with ureteral stent placement. *BMJ Case Rep.* **2018**, *2018*, bcr2017222908. https://doi.org/10.1136/bcr-2017-222908.
218a. Nardone, R.; Venturi, A.; Buffone, E.; Lochner, P.; Marth, R.; Florio, I.; Psenner, K.; Tezzon, F. Extramedullary Spinal Neurosarcoidosis: Report of Two Cases. *Eur. Neurol.* **2005**, *54*, 220–224. https://doi.org/10.1159/000090714.
219a. Narula, N.; Ngu, S.; Avula, A.; Mansour, W.; Chalhoub, M. Left-sided Catamenial Pneumothorax: A Rare Clinical Entity. *Cureus* **2018**, *10*, e2567. https://doi.org/10.7759/cureus.2567.
220a. Nazir, S.; Melnick, S.; Lohani, S.; Lloyd, B. Rare case of stress cardiomyopathy due to intramuscular epinephrine administration. *BMJ Case Rep.* **2016**, *2016*, bcr2016215691. https://doi.org/10.1136/bcr-2016-215691.
221a. Newcomb, A.E.; Clarke, C.P. Spontaneous pneumomediastinum: A benign curiosity or a significant problem?. *Chest* **2005**, *128*, 3298–3302.
222a. Newman, W.C.; Williams, L.; Duvvuri, U.; Clump, D.A.; Amankulor, N. Hyalinizing Clear Cell Carcinoma with Biopsy-Proven Spinal Metastasis: Case Report and Review of Literature. *World Neurosurg.* **2016**, *90*, 699.e7–699.e10. https://doi.org/10.1016/j.wneu.2016.02.001.
223a. Ng, N.Y.B.; Twoon, M.; E. Thomson, S. Psoas abscess and severe fasciitis due to a caecal carcinoma. *BMJ Case Rep.* **2015**, *2015*, bcr2014207494. https://doi.org/10.1136/bcr-2014-207494.
224a. Nguyen J., Tyson C., Glass M. Celiac Curveball. *J. Hosp. Med.* **2011**, *6* (Suppl 2.), 351. Available online: https://shmabstracts.org/abstract/celiac-curveball/ (accessed on 4 May 2022).
225a. Noël, P.; Preux, G.; Thézé, A. Epidural lipomatosis: A possible cause of back pain. *Ann. Phys. Rehabilitation Med.* **2014**, *57*, 734–737. https://doi.org/10.1016/j.rehab.2014.08.001.
226a. O'Callaghan, D.S.; Breen, D.P.; Young, V. Angiosarcoma of the Right Atrium Masquerading as Recurrent Pulmonary Embolism. *Thorac. Cardiovasc. Surg.* **2008**, *56*, 488–490. https://doi.org/10.1055/s-2007-989292.
227a. Oh, Y.; Kim, J.-T.; Kim, M.-K.; Chang, Y.-J.; Eom, K.; Park, J.-G.; Lee, K.-M.; Choe, K.-H.; An, J.-Y. Eosinophilic Pleuritis due to Sparganum: A Case Report. *Korean J. Parasitol.* **2014**, *52*, 541–543. https://doi.org/10.3347/kjp.2014.52.5.541.
228a. Oike, N.; Kawashima, H.; Ogose, A.; Hotta, T.; Hirano, T.; Ariizumi, T.; Yamagishi, T.; Umezu, H.; Inagawa, S.; Endo, N. A malignant solitary fibrous tumour arising from the first lumbar vertebra and mimicking an osteosarcoma: A case report. *World J. Surg. Oncol.* **2017**, *15*, 100. https://doi.org/10.1186/s12957-017-1161-0.
229a. Onur, O.O.; Guneysel, O.; Saritemur, M.; Denizbasi, A. Spontaneous rupture of hepatocellular carcinoma presented as low back pain to an emergency department: A case report. *BMJ Case Rep.* **2009**, *2009*, bcr0720080580. https://doi.org/10.1136/bcr.07.2008.0580.

230a. Ors, F.; Deniz, O.; Bozlar, U.; Ozcan, A. Coexistent pulmonary tuberculosis, endometrial tuberculosis and intracranial tuberculomas in a non-immunocompromised woman. *Anatol. J. Clin. Investig.* **2008**, *2*, 123–126.
231a. Osken, A.; Aydın, E.; Kocayigit, I.; Cakar, M.; Gündüz, H.; Yaylacı, S.; Tamer, A. Slow ventricular response atrial fibrillation related to mad honey poisoning. *J. Cardiovasc. Dis. Res.* **2012**, *3*, 245–247. https://doi.org/10.4103/0975-3583.98904.
232a. Ozsarac, M.; Karcioglu, O.; Ayrik, C.; Bozkurt, S.; Turkcuer, I.; Gumrukcu, S. Red flag in the Emergency Department: Fracture and primary failure of a prosthetic valve. *J. Emerg. Med.* **2005**, *29*, 49–51. https://doi.org/10.1016/j.jemermed.2005.01.006.
233a. zturan İU, Köse B., Özkan B., Köse A. Myopericarditis caused by severe acute respiratory syndrome coronavirus 2. *Clin. Exp. Emerg Med.* **2020**, *7*, 326–329.
234a. Pai, S.; Panda, M. Limited Wegener's granulomatosis presenting as lung nodules in a patient with rheumatoid arthritis: A case report. *Cases J.* **2008**, *1*, 417–4,. https://doi.org/10.1186/1757-1626-1-417.
235a. Panjwani, A.; Zaid, T. An interesting case of undiagnosed pleural effusion. *Breathe* **2017**, *13*, e46–e52. https://doi.org/10.1183/20734735.001117.
236a. Parikh, S.A.; French, C.A.; Costello, B.A.; Marks, R.S.; Dronca, R.S.; Nerby, C.L.; Roden, A.C.; Peddareddigari, V.G.; Hilton, J.; Shapiro, G.I.; et al. NUT Midline Carcinoma: An Aggressive Intrathoracic Neoplasm. *J. Thorac. Oncol.* **2013**, *8*, 1335–1338. https://doi.org/10.1097/jto.0b013e3182a00f41.
237a. Park, M.J.; Sperber, K. Piriformis Abscess—A Rare Cause of Sciatica: A Case Report. *PM&R* **2013**, *5*, S300. https://doi.org/10.1016/j.pmrj.2013.08.543.
238a. Park, S.H.; Choi, S.B.; Kim, W.B.; Song, T.-J. Huge primary osteosarcoma of the liver presenting an aggressive recurrent pattern following surgical resection. *J. Dig. Dis.* **2009**, *10*, 231–235. https://doi.org/10.1111/j.1751-2980.2009.00391.x.
239a. Patel, S.; Agrawal, A.; Maheshwari, R.; Chauhan, V.D. Periosteal Osteoblastoma of the Pelvis: A Rare Case. *Iran J. Med. Sci.* **2015**, *40*, 77–80.
240a. Patel, S.R.; Ammar, A.; Khan, R.A.; Weatherly, M.R. A Triple Threat: Acute Pulmonary Emboli, Pneumococcal Pneumonia and Congenital Bronchiectasis with Auto-Pneumonectomy and Compensatory Hyper-Lobar Expansion. *Am. J. Respir. Crit. Care Med.* **2019**, *199*, A6450
241a. Pechman, D.M.; Bonati, L.M.; Harrington, M.D. Painful Anterior Chest Wall Mass, Compatible With Abscess on Computed Tomography and Possible Osteomyelitis in a Man With Ulcerative Colitis. *Gastroenterology* **2016**, *151*, 398–399. https://doi.org/10.1053/j.gastro.2016.04.042.
242a. Pedrazas López, D.; de Pablo Márquez, B.; García Font, D.; Alins Presas, J.; Esplugas Abòs, J. Sarcoma endoluminal. *Gac Mex Oncol.* **2016**, *15*, 254–257.
243a. Spencker, S.; Hohmann, C.; Mochmann, H.C. Cerebral ischemia and pulmonary embolism with riding thrombus. *J. Emerg Med.* **2010**, *38*, 656–657.
244a. Peng, H.; Chen, W.-F.; Wu, C.; Chen, Y.-R.; Peng, B.; Paudel, S.D.; Lou, T.-Q. Culture-negative subacute bacterial endocarditis masquerades as granulomatosis with polyangiitis (Wegener's granulomatosis) involving both the kidney and lung. *BMC Nephrol.* **2012**, *13*, 174–174. https://doi.org/10.1186/1471-2369-13-174.
245a. Penkulinti, M.; Raorane, H.; Kalakata, V.S.; Vemuri, S.K.; Banala, R.R.; Gpv, S. Enchondroma Protuberans of the Transverse Process of D8 Vertebra Extending to the 7th and 8th Ribs: A Rare Case Report. *Int. J. Spine Surg.* **2018**, *12*, 43–48. https://doi.org/10.14444/5008.
246a. Perrin, A.; Bergman, C.; Hart, L. A 30-Year-Old Industrial Worker With Upper Back Pain. *Adv. Emerg. Nurs. J.* **2013**, *35*, 103–109. https://doi.org/10.1097/tme.0b013e3182948e73.
247a. Petersen, D.; Lystad, R.P. Spinal myxopapillary ependymoma in an adult male presenting with recurrent acute low back pain: A case report. *Chiropr. Man. Ther.* **2016**, *24*, 11. https://doi.org/10.1186/s12998-016-0094-y.
248a. Plymyer, M.R.; Rice, M.R. A Young Woman Presenting with Acute Onset of Chest Pain. *Lab Med.* **2003**, *34*, 274–276.
249a. Poddar, N.; Avezbakiyev, B.; He, Z.; Jiang, M.; Gohari, A.; Wang, J.C. Hepatocellular Carcinoma Presenting as an Incidental Isolated Malignant Portal Vein Thrombosis. *J. Gastrointest. Cancer* **2010**, *43*, 486–489. https://doi.org/10.1007/s12029-010-9235-z.
250a. Przybojewski, J.Z. Recurrent coronary artery spasm in the billowing mitral leaflet syndrome (primary mitral valve prolapse). A case report and review of the literature. *S. Afr. Med. J.* **1984**, *66*, 138–143.
251a. Pytel, P.; Rezania, K.; Soliven, B.; Frank, J.; Wollmann, R. Chronic inflammatory demyelinating polyradiculoneuropathy (CIDP) with hypertrophic spinal radiculopathy mimicking neurofibromatosis. *Acta Neuropathol.* **2003**, *105*, 185–188. https://doi.org/10.1007/s00401-002-0616-7.
252a. Rahim, A.H.; Hidajat, N.N.; Ramdan, A.; Magetsari, R.M.S.N. Fibrous Dysplasia of the Spine—A Case Involving the Polyostotic Form Isolated to the Thoracolumbar Spine. *Int. J. Spine Surg.* **2020**, *14*, S46–S51. https://doi.org/10.14444/7164.
253a. Rahmani, R.; Parsa, A.Z.; Sherafati, A.; Kosari, R.; Mohhamadi, V.; Mohhamadi, R. A Rare Case of Cardiogenic Shock following Severe Multivessel Coronary Vasospasm. *J. Tehran Univ. Heart Cent.* **2019**, *14*, 28–32. https://doi.org/10.18502/jthc.v14i1.653.
254a. Rajagopala, S.; Devaraj, U.; D'Souza, G. Infected Cavitating Pulmonary Infarction. *Respir. Care* **2011**, *56*, 707–709. https://doi.org/10.4187/respcare.00828.
255a. Rajasekharan, C.; Deepak, M.; Ratheesh, N.S. Fatal haemoptysis due to ruptured aortic arch aneurysm with aorto-pulmonary fistula. *BMJ Case Rep.* **2012**, *2012*, bcr2012006946. https://doi.org/10.1136/bcr-2012-006946.
256a. Ranu, H.; Buyck, H.; Willis, F.; Madden, B. Breathlessness and chest pain in a patient with sickle cell disease. *BMJ* **2011**, *343*, d3797. https://doi.org/10.1136/bmj.d3797.
257a. Rao, L.; Kudva, R.; Rao, R.V.; Kumar, B. Extraskeletal Myxoid Chondrosarcoma of the Chest Wall Masquerading as a Breast Tumor. *Acta Cytol.* **2002**, *46*, 417–421. https://doi.org/10.1159/000326743.

258a. Rapaport, E. A nonclassic case of pulmonary embolism. *Hosp. Pract. (Off. Ed).* **1992**, *27*, 86–102.
259a. Reddy, H.R.; Yick, D.G.; Kamangar, N. A Case of Primitive Neuroectodermal Tumor (Pnet) of the Chest Wall in an Adult. *Chest* **2009**, *136*, 32S–f-33S. https://doi.org/10.1378/chest.136.4_meetingabstracts.32s-f.
260a. Robertson, E.K.; Evans, A.L. Lower Thoracic Spine Pain in a 33-Year-Old Female. *J. Orthop. Sports Phys. Ther.* **2010**, *40*, 533. https://doi.org/10.2519/jospt.2010.0412.
261a. Roche-Nagle, G.; Rubin, B.B. Considerations in the diagnosis and therapy for acute loin pain. *Am. J. Emerg. Med.* **2009**, *27*, 254.e3–254.e4. https://doi.org/10.1016/j.ajem.2008.06.030.
262a. Rohilla, R.; Meena, S.; Kaistha, N.; Raj, A.K.; Gupta, P. Disseminated cryptococcosis and hepatitis C virus infection: A fatal co-infection. *Curr. Med. Mycol.* **2020**, *5*, 50–53. https://doi.org/10.18502/cmm.5.4.2163.
263a. Ross, M.D.; Bayer, E. Cancer as a Cause of Low Back Pain in a Patient Seen in a Direct Access Physical Therapy Setting. *J. Orthop. Sports Phys. Ther.* **2005**, *35*, 651–658. https://doi.org/10.2519/jospt.2005.35.10.651.
264a. Rovlias, A.; Balanika, A.; Nomikos, A.; Melissaris, S. Primary Myxoid Liposarcoma of the Upper Thoracic Spine in an Elderly Patient. *J. Neurosci. Rural Pract.* **2017**, *8*, S120–S122. https://doi.org/10.4103/jnrp.jnrp_99_17.
265a. Rubin, R.N. Radiating midsternal pain in a middle-aged woman. *Consultant* **2004**, *44*, 1441–1444.
266a. Safia, R.; Muqim R ul Raza, H.; Muhammad, Z.; Mohammad Aziz, W.; Mahmud, A. Inferior lumbar hernia of petit in a patient with neurofibromatosis type-1. *Pak. J. Med. Sci.* **2009**, *25*, 1015–1017.
267a. Saha, D.; Selema, D.; Leon, J.; Gottlieb, S. Atypical Presentation of Community Acquired Pseudomonas Pneumonia in a Healthy Individual. *Chest* **2014**, *146*, 168A. https://doi.org/10.1378/chest.1994251.
268a. Salehpour, F.; Mirzaei, F.; Rezakhah, A.; Aeinfar, K.; Kazemzadeh, M.; Alavi, S.A.N. Ligamentum Flavum Hematoma Presented with Low Back Pain: A Case Report and Review of the Literature. *Int. J. Spine Surg.* **2018**, *12*, 337–341. https://doi.org/10.14444/5039.
269a. Sawayama, T.; Shiozu, N.; Niki, I.; Matsuura, T.; Ichinose, S. Unusual Form of Impending Myocardial Infarction in a Premenopausal Woman. *Jpn. Circ. J.* **1965**, *29*, 943–950. https://doi.org/10.1253/jcj.29.943.
270a. Schoeters, P.; De Raedt, H.; Colemont, L. An unusual case of biventricular cardiac failure. *Acta Cardiol.* **2001**, *56*, 127–129. https://doi.org/10.2143/AC.56.2.2005629.
271a. Schultz, J.C.; Lassi, N.K.; Edson, R.S. 19-Year-Old Man With Chest Pain, Fever, and Vomiting. *Mayo Clin. Proc.* **2007**, *82*, 1405–1408. https://doi.org/10.4065/82.11.1405.
272a. Seferi, A.; Alimehmeti, R.; Vyshka, G.; Bushati, T.; Petrela, M. Case Study of a Spinal Epidural Capillary Hemangioma: A 4-Year Postoperative Follow-Up. *Glob. Spine J.* **2013**, *4*, 055–058. https://doi.org/10.1055/s-0033-1354251.
273a. Selvaratnam, R.; Srirangalingam, U.; Mclean, E.; Lang-Lazdunski, L.; Goulden, P. A rare cause of acute chest pain in a young adult. *Clin. Med.* **2011**, *11*, 265–267. https://doi.org/10.7861/clinmedicine.11-3-265.
274a. Shah, E.V.; Francis, S.A.; Shah, A.D.; Shah, K.V. Pancreaticopleural fistula masquerading as hemorrhagic pleural effusion. *Lung India* **2018**, *35*, 454–455. https://doi.org/10.4103/lungindia.lungindia_81_18.
275a. Shah, R.P.; Lora, M.H. A hole in our logic: Using bayesian probabilities to overcome anchoring bias. *J. Gen. Int. Med.* **2019**, *3*, S443–S444.
276a. Shankarappa, R.K.; Moorthy, N.; Chandrasekaran, D.; Nanjappa, M.C. Giant pulmonary artery aneurysm secondary to primary pulmonary hypertension. *Tex. Heart Inst. J.* **2010**, *37*, 244–245.
277a. Shantanu, K.; Sharma, V.; Kumar, S.; Jain, S. Tuberculosis of sacrum mimicking as malignancy. *BMJ Case Rep.* **2012**, *2012*, bcr0720114505. https://doi.org/10.1136/bcr.07.2011.4505.
278a. Sharma, T.; Tigadi, S.M.; Baldwin, J.; Tabtabai, S.R. Clinical Overlap Between Myopericarditis and Stress Induced Cardiomyopathy: A Diagnostic and Therapeutic Challenge. *Am. J. Case Rep.* **2019**, *20*, 252–257. https://doi.org/10.12659/ajcr.912169.
279a. Shian, B.; Eken, J. Chest pain with diffuse T-wave inversion. *Am. Fam. Physician* **2014**, *90*, 57–258.
280a. Shields, D.W.; Robinson, P.G. Iliopsoas abscess masquerading as "sciatica." *Case Rep.* **2012**, *2012*, bcr2012007419.
281a. Shim, H.-K.; Cho, H.-L.; Lee, S.-H. Spinal tuberculosis at the posterior element of spinal column: Case report. *Clin. Neurol. Neurosurg.* **2014**, *124*, 146–150. https://doi.org/10.1016/j.clineuro.2014.05.021.
282a. Shivji, F.; Tsegaye, M. Cauda equina syndrome: The importance of complete multidisciplinary team management. *BMJ Case Rep.* **2013**, *2013*, bcr2012007806. https://doi.org/10.1136/bcr-2012-007806.
283a. Shogbesan, O.; Jehangir, A.; Abdulkareem, A.; Byreddy, S.; Donato, A. Testicular Germ Cell Tumor Presenting With Occult Gastrointestinal Bleeding. *Am. J. Gastroenterol.* **2017**, *112*, S1035. https://doi.org/10.14309/00000434-201710001-01878.
284a. Som, R.; Li, A.; McIntosh, R.; Lloyd, G. Acute dyspnoea, dysphagia, and non-specific chest pain in a smoker. *BMJ* **2008**, *337*, a2678. https://doi.org/10.1136/bmj.a2678.
285a. Sonawane, D.V.; Jagtap, S.A.; Patil, H.G.; Biraris, S.R.; Chandanwale, A.S. Intramedullary Tuberculoma of Dorsal Spinal Cord: A Case Report with Review of Literature. **2016**, *5*, 44–46. https://doi.org/10.13107/jocr.2250-0685.271.
286a. Song, L.; Bian, G.; Yang, W.; Li, H.-F. Variant angina induced by carbon monoxide poisoning. *Medicine* **2019**, *98*, e15056. https://doi.org/10.1097/md.0000000000015056.
287a. Southerst, D.; Dufton, J.; Stern, P. Multiple Myeloma presenting as sacroiliac joint pain: A case report. *J. Can. Chiropr. Assoc.* **2012**, *56*, 94–101.
288a. Spece, L.J.; Reder, N.P.; Gibbons, E.F.; West, T.E. Gemella Endocarditis Associated with a Congenital Bicuspid Aortic Valve. *Am. J. Respir. Crit. Care Med.* **2015**, *191*, A1672.

289a. Stross, W.C.; Alsaad, A.A.; Shapiro, B.P. 42-Year-Old Woman With Bilateral Arm Tightness. *Mayo Clin. Proc.* **2018**, *93*, e45–e51. https://doi.org/10.1016/j.mayocp.2017.05.032.
290a. Subramanian, S.; Jonathan, G.E.; Patel, B.; Prabhu, K. Synovial sarcoma mimicking a thoracic dumbell schwannoma- a case report. *Br. J. Neurosurg.* **2018**, *34*, 98–101. https://doi.org/10.1080/02688697.2017.1418289.
291a. Suen, P.; Siddiqui, A.; Catella, P.; Wiesel, S.; Chalhoub, M. Dysphagia as a Presentation of Spontaneous Mediastinal Hematoma With Life-Threatening Airway Obstruction. *Chest* **2017**, *152*, A495. https://doi.org/10.1016/j.chest.2017.08.523.
292a. Suzuki, K.; Yasuda, T.; Hori, T.; Watanabe, K.; Kanamori, M.; Kimura, T. An intraosseous malignant peripheral nerve sheath tumor of the lumbar spine without neurofibromatosis: Case report and review of the literature. *Oncol. Lett.* **2014**, *7*, 1965–1969. https://doi.org/10.3892/ol.2014.1987.
293a. Tamae Kakazu MA., Clapp W., Patel A. Actinobacillus Actinomycetemcomitans Causing empyema Necessitatis. Am J Respir Crit Care Med. **2010**, *181*, A4734.
294a. Tandan, N.; Liao, H.-I.; Adarkwah, O. A curious case of myocarditis with elevated antistreptolysin o titers. *Chest* **2019**, *156*, A399. https://doi.org/10.1016/j.chest.2019.08.436.
295a. Tekçe, H.; Aktaş, G.; Öztürk, S. A distal (type 1) renal tubular acidosis case that mimic coronary ischemia. *Ren. Fail.* **2013**, *35*, 1289–1291. https://doi.org/10.3109/0886022x.2013.820662.
296a. Teo, K.a.T.; Burns, E.; Garcea, G.; Abela, J.E.; McKay, C.J. Incarcerated small bowel within a spontaneous lumbar hernia. *Hernia J. Hernias Abdom Wall Surg.* **2010**, *14*, 539–541.
297a. Ter Horst, M.; Stam, M.A.W.; Bouman, D.E.; Klaase, J.M. Cauda Equina Syndrome Secondary to Complicated Diverticulitis. *Case Rep. Gastroenterol.* **2013**, *7*, 455–461.
298a. Timmerman, M.A.; Fredericson, M. Poster 225 Mid-Back Pain in a 28-Year-Old Male Rower Presenting to the Sports Medicine Clinic: An Atypical Presentation of Cushing's Disease. A Case Report. *PM&R* **2012**, *4*, S266–S267. https://doi.org/10.1016/j.pmrj.2012.09.844.
299a. Tokue, M.; Hara, H.; Kurosawa, K.; Nakamura, M. Fulminant myocardial bleeding: Another clinical course of vascular Ehlers-Danlos Syndrome. *BMJ Case Rep.* **2017**, *2017*, bcr-2017-220786. https://doi.org/10.1136/bcr-2017-220786.
300a. Toujani, S.; Abida, R.; El Ouni, A.; Belhassen, A.; Abdelkefi, C.; Meddeb, Z.; Larbi, T.; Hamzaoui, S.; Bouslama, K. Pheochromocytoma presenting as an authentic small vessel vasculitis and complicated with pulmonary embolism: An original presentation. *Annales de Cardiologie et d'Angéiologie* **2021**, *70*, 168–170. https://doi.org/10.1016/j.ancard.2021.04.006.
301a. Tsang, W.; Li, A. Coronary Artery Aneurysm with Associated Fistula: A Rare Condition Detected by Computed Tomography Coronary Angiography. *Hong Kong J. Radiol.* **2014**, *17*, 57–61. https://doi.org/10.12809/hkjr1412123.
302a. Ünal O, Özçakar L, İnanıcı F. Magnetic resonance imaging: A sine qua non in the diagnosis of brucella spondylitis. *Clin Rheumatol.* **2004**, *23*, 473–474.
303a. Unnikrishnan, D.; DeLaCruz, M.A.; Saha, A.; Daniels, R. Case of acute paraspinal pyomyositis in an elderly diabetic secondary to spread from urinary tract infection. *BMJ Case Rep.* **2018**, *2018*, bcr-2018. https://doi.org/10.1136/bcr-2018-225739.
304a. Van Tankeren, E.; Desmedt, H.; Van Der Mieren, G.; Somville, F. Spontaneous Pseudomediastinum. *Acta Clin Belg.* **2020**, *75*, 35.
305a. Varshney, M.K.; Trikha, V.; Alam Khan, S. Actinomycosis or tuberculosis? A diagnostic dilemma. *Scand. J. Infect. Dis.* **2006**, *38*, 378–381. https://doi.org/10.1080/00365540500447168.
306a. Verlage, K.R.; Underwood, L.; de Riese, W.T.W. Retroperitoneal metastatic germ cell tumor presenting as a psoas abscess: A rare clinical occurrence and review of the literature. *Int. Urol. Nephrol.* **2016**, *48*, 1839–1840. https://doi.org/10.1007/s11255-016-1385-x.
307a. Wang, S.-J.; Cao, D.-L.; Xu, H.-W.; Zhao, W.-D.; Hu, T.; Wu, D.-S. Development of Primary Diffuse Large B-Cell Lymphoma Around an Internal Fixation Implant After Lumbar Fusion Surgery: A Case Report and Review of the Literature. *World Neurosurg.* **2020**, *137*, 140–145. https://doi.org/10.1016/j.wneu.2020.01.227.
308a. Widyawati, D.G.; Rina, K. Acute fulminant myocarditis mimicking ST-elevation myocardial infarction. *Eur. Heart J. Suppl.* **2016**, *18*, b51.
309a. Xin, L.; Wang, Z.; Fan, S. Magnetic resonance imaging and computerised tomography findings in an intraspinal extradural hydatid cyst mimicking tuberculous spondylitis: A case report. *Cases J.* **2009**, *2*, 7109. https://doi.org/10.4076/1757-1626-2-7109.
310a. Yang, T.-Y.; Cheng, N.-J.; Chang, C.-J.; Lai, M.-C.; Ko, Y.-L. Giant coronary artery aneurysm mimicking a paracardiac mass. *Chang Gung Med. J.* **2003**, *26*, 133–137.
311a. Yemisci, O.U.; Cosar, S.N.S.; Oztop, P.; Karatas, M. Spondylodiscitis associated with multiple level involvement and negative microbiological tests: An unusual case. *Spine* **2010**, *35*, E1006–E1009.
312a. Younus, U.; Abbott, B.; Narasimha, D.; Page, B.J. Coronary Subclavian Steal Syndrome: An Unusual Cause of Angina in a Post-CABG Patient. *Case Rep. Cardiol.* **2014**, *2014*, 769273. https://doi.org/10.1155/2014/769273.
313a. Zapolski, T.; Mariusz Kozak, M.K.; Agnieszka Styczen, A.S.; Marta Karas Glodek, M.K.G.; Andrzej Tomaszewski, A.T.; Elzbieta Czekajska Chehab, E.C.C. 1116 Atypical, cardiac location of melanoma. *Eur. Heart J. Cardiovasc. Imaging* **2020**, *21*, jez319.661.
314a. Zarabi, C.M.; Huntrakoon, M.; Fine, K.D. Disseminated Rhabdomyosarcoma of the Urinary Bladder in an Adult. *South. Med. J.* **1987**, *80*, 526–529. https://doi.org/10.1097/00007611-198704000-00031.
315a. Zhang, P.; Meng, X.; Xia, L.; Xie, P.; Sun, X.; Gao, Y.; Wang, S.; Zhao, X.; Yu, J. Non-small cell lung cancer with concomitant intramuscular myxoma of the right psoas mimicking intramuscular metastasis: A case report and literature review. *Oncol. Lett.* **2015**, *10*, 3059–3063. https://doi.org/10.3892/ol.2015.3704.

316a. Zhang, T.; Peng, B.; Tu, X.; Zhang, S.; Zhong, S.; Cao, W. Acute myocardial infarction as the first manifestation of Takayasu arteritis. *Medicine* **2019**, *98*, e15143. https://doi.org/10.1097/md.0000000000015143.
