# Supplementary material for: Screening for Referral of Serious Pathology by Physical Examination Tests in Patients with Back or Chest Pain: A Systematic Review
Source: Int J Environ Res Public Health. 2022 Dec 7;19(24):16418. doi: 10.3390/ijerph192416418 (PMC9779291; doi:10.3390/ijerph192416418)
Supplement: Supplementary file 1 [file ijerph-19-16418-s001.zip › Supplementary S4 for XML.pdf]

Supplementary S4

The bibliographic list of 26 studies in which clinicians declared the suspicion of serious disease at the end of the physical examination

References

4a. Ahmad, I.; Goyal, N.; Bhatt, C.P.; Chufal, K.S. Primary vertebral leiomyosarcoma masquerading as a nerve sheath tumour. *BMJ Case Rep.* **2017**, *2017*, bcr2016217602. https://doi.org/10.1136/bcr-2016-217602.

5a. Ahmed, A.; Garba, I.; Denue, B.A.; Alkali, M.B.; Bakki, B.; Rawizza, H. Catamenial right haemothorax due to endometriosis: Two case reports. *Afr. J. Respir.* **2015**, *10*, 24–26.

6a. Belliveau, D.J.; Weeks, A.C.; McKelvey, R.; Mulvagh, S.L. Cardiogenic Shock in a Healthy Young Woman With Acute Onset Abdominal and Chest Pain. *JACC: Case Rep.* **2020**, *2*, 24–27. https://doi.org/10.1016/j.jaccas.2019.11.066.

7a. Boissonnault, W.G.; Thein-Nissenbaum, J.M. Differential Diagnosis of a Sacral Stress Fracture. *J. Orthop. Sports Phys. Ther.* **2002**, *32*, 613–621. https://doi.org/10.2519/jospt.2002.32.12.613.

8a. Cabanelas, N.; Nobre, Â.; Guerra, N.; Gallego, J.; Ferreira, R.; Carvalheiro, C.; Roque, J.; Peres, M.; Siopa, L.; Martins, V.P.; et al. Images in Cardiology after clinical observation—Aortic dissection in Marfan syndrome. *Rev. Port. De Cardiol.* **2011**, *30*, 735–741.

9a. Campos, I.D.; Salgado, A.; Azevedo, P.; Vieira, C. Dressler's syndrome: Are we underdiagnosing what we think to be rare? *BMJ Case Rep.* **2019**, *12*, e227772. https://doi.org/10.1136/bcr-2018-227772.

10a. Christe, G.; Hall, T. The screening process of a patient with low back pain and suspected thoracic myelopathy: A case report. *J. Man. Manip. Ther.* **2017**, *26*, 11–17. https://doi.org/10.1080/10669817.2017.1282189.

11a. Crowell, M.S.; Gill, N.W. Medical Screening and Evacuation: Cauda Equina Syndrome in a Combat Zone. *J. Orthop. Sports Phys. Ther.* **2009**, *39*, 541–549. https://doi.org/10.2519/jospt.2009.2999.

12a. Erhard, R.E.; Egloff, B.P. Patient With Metastatic Adenocarcinoma Imitating Lumbar Herniated Nucleus Pulposis. *J. Manip. Physiol. Ther.* **2004**, *27*, 569–573. https://doi.org/10.1016/j.jmpt.2004.10.001.

13a. Gray, J.C. Diagnosis of Intermittent Vascular Claudication in a Patient With a Diagnosis of Sciatica. *Phys. Ther.* **1999**, *79*, 582–590. https://doi.org/10.1093/ptj/79.6.582.

14a. Greene, G. "Red Flags": Essential factors in recognizing serious spinal pathology. *Man. Ther.* **2001**, *6*, 253–255.

15a. Greenwood, M.J.; Erhard, R.E.; Jones, D.L. Differential diagnosis of the hip vs. lumbar spine: Five case reports. *J. Orthop. Sport. Phys. Ther.* **1998**, *27*, 308–315.

16a. Harris, K.K.; Delic, J.A.; Nelson, E.O. Epidural and Paraspinal Abscess Presenting as Acute Low Back Pain.. *J. Orthop. Sports Phys. Ther.* **2019**, *49*, 482–482. https://doi.org/10.2519/jospt.2019.8456.

17a. Kaufman, R. Early-Onset Multiple Myeloma: An Illustrative Case Report. *J. Manip. Physiol. Ther.* **2007**, *30*, 543–549. https://doi.org/10.1016/j.jmpt.2007.07.002.

18a. Kodaira, M.; Itoh, T.; Koizumi, K.; Numasawa, Y. Giant right coronary artery aneurysm complicated by a fistula to the right atrium, mimicking a ruptured sinus of Valsalva aneurysm. *BMJ Case Rep.* **2018**, *2018*, bcr2018226197. https://doi.org/10.1136/bcr-2018-226197.

19a. Laslett, M. Bilateral buttock pain caused by aortic stenosis: A case report of claudication of the buttock. *Man. Ther.* **2000**, *5*, 227–233. https://doi.org/10.1054/math.2000.0368.

20a. Lyon, M.; Brannam, L.; Ciamillo, L.; Blaivas, M. False positive abdominal aortic aneurysm on bedside emergency ultrasound. *J. Emerg. Med.* **2004**, *26*, 193–196. https://doi.org/10.1016/j.jemermed.2003.08.012.

21a. Martinez, M.; Lee, A.S.; Hellinger, W.C.; Kaplan, J. Vertebral Aspergillus Osteomyelitis and Acute Diskitis in Patients With Chronic Obstructive Pulmonary Disease. *Mayo Clin. Proc.* **1999**, *74*, 579–583. https://doi.org/10.4065/74.6.579.

22a. Maselli, F.; Rossettini, G.; Viceconti, A.; Testa, M. Importance of screening in physical therapy: Vertebral fracture of thoracolumbar junction in a recreational runner. *BMJ Case Rep.* **2019**, *12*, e229987. https://doi.org/10.1136/bcr-2019-229987.

23a. Matsumura, A.; Ayuzawa, S.; Doi, M.; Enomoto, T.; Takeuchi, S.; Yoshii, Y.; Nose, T. Chronic progressive hematomyelia: Case reports and review of the literature. *Surg. Neurol.* **1999**, *51*, 559–563. https://doi.org/10.1016/s0090-3019(97)00442-4.

24a. McAree, B.J.; E. O'Donnell, M.; Boyd, C.; Spence, R.A.; Lee, B.; Soong, C.V. Inferior Vena Cava Thrombosis in Young Adults—A review of two cases. *Ulster Med. J.* **2009**, *78*, 129–133.

25a. Panwar, N.; Purohit, D.K.; Sharma, S.; Chopra, S. Symptomatic Thoracic Intramedullary Arachnoid Cyst: "A Rare Entity" Report of Two Cases with Short Review of Literature. *J. Neurosci. Rural. Pract.* **2019**, *10*, 306–311. https://doi.org/10.4103/jnrp.jnrp_246_18.

26a. Ross, M.D.; Elliott, R.L. Thoracic Spine Compression Fracture in a Patient With Back Pain. *J. Orthop. Sports Phys. Ther.* **2008**, *38*, 214–214. https://doi.org/10.2519/jospt.2008.0404.

27a. Roxas, R.; Gallegos, L.; Bailitz, J. Rapid Detection of Aortic Occlusion With Emergency Ultrasonography. *Ann. Emerg. Med.* **2011**, *58*, 21–23. https://doi.org/10.1016/j.annemergmed.2010.09.001.

28a. Stowell, T.; Cioffredi, W.; Greiner, A.; Cleland, J. Abdominal Differential Diagnosis in a Patient Referred to a Physical Therapy Clinic for Low Back Pain. *J. Orthop. Sports Phys. Ther.* **2005**, *35*, 755–764. https://doi.org/10.2519/jospt.2005.35.11.755.

29a. Tzeng, C.-H.; Chen, W.-K.; Lu, H.-C.; Chen, H.-H.; Lee, K.-I.; Wu, Y.-S.; Lee, F.-Y. Challenges in the diagnosis of Boerhaave syndrome. *Medicine* **2020**, *99*, e18765. https://doi.org/10.1097/md.0000000000018765.
